# Supplementary material for: Phylogenetic analysis reveals an ancient gene duplication as the origin of the MdtABC efflux pump
Source: PLoS One. 2020 Feb 12;15(2):e0228877. doi: 10.1371/journal.pone.0228877 (PMC7015380; doi:10.1371/journal.pone.0228877)
Supplement: S1 File — (DOCX) [file pone.0228877.s001.docx]

Supplementary material

# Horizontal Gene Transfer

## *Rhodopseudomonas palustris* strains

As described in the main text, the two *Rhodopseudomonas palustris* strains differ in their single subunit RND systems. The CGA009 strain looks like its close relatives from other Alphaproteobacteria (an MdtBC and an MexN), and the HaA2 strain contains a “progenitor-like” RND. The Supplementary Figure 1 below shows the two locations in their genomes where the corresponding deletion and insertion occurred.


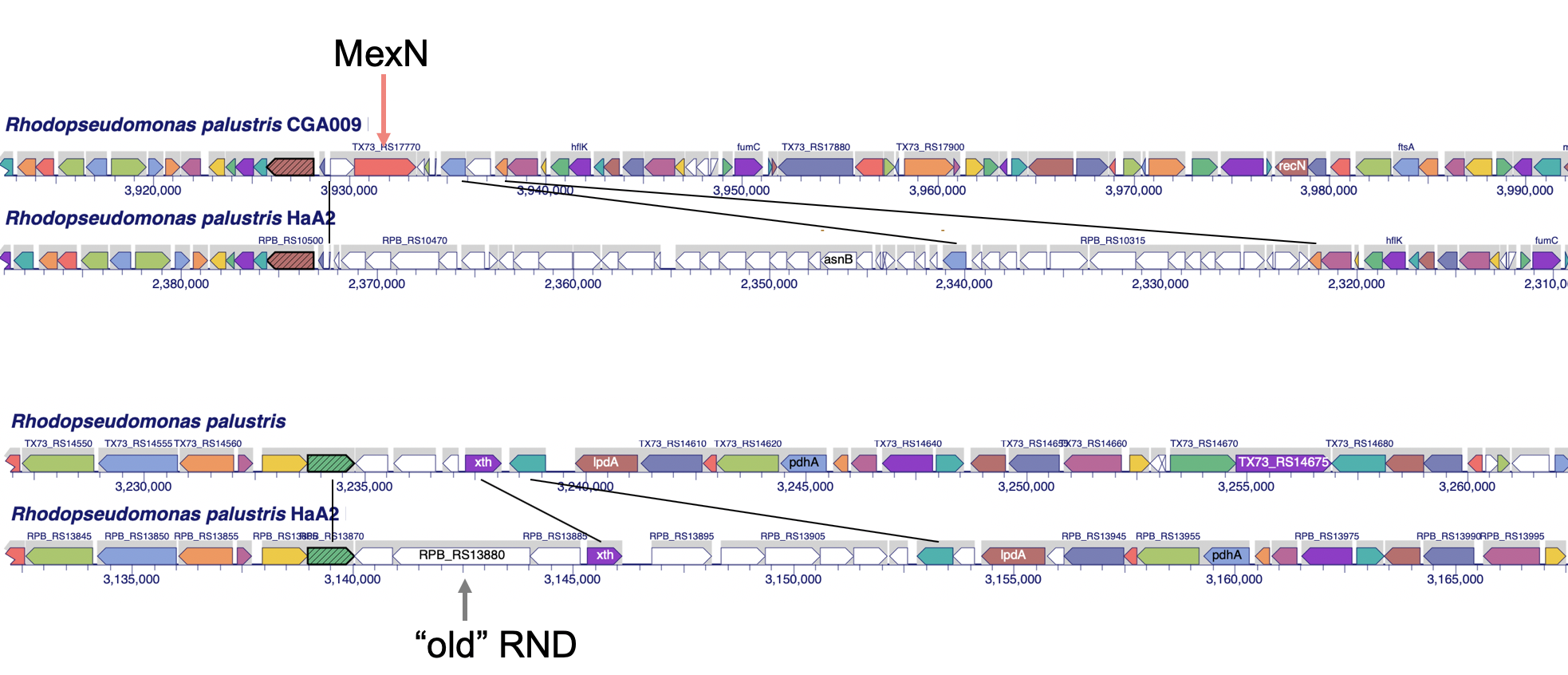


Supplementary Figure 1. Alignment of genomes of the two strains of Rhodopseudomonas palustris. Upper panel, a spot where the MexN was lost in the strain HaA2. Lower panel, an insertion point of the “progenitor-like” RND.

Translation MNMSRLFILRPVATTLSMLAIVLAGLIAYTLLPVSALPQVDYPTIRVMTLYPGASPQVMT     60

PSEU2 MdtB      MNMSRLFILRPVATTLSMLAIVLAGLIAYTLLPVSALPQVDYPTIRVMTLYPGASPQVMT     60

************************************************************

Translation SSVTAPLERQFGQMPGLTQMASTSSGGASVITLRFSLEINMDVAEQQVQAAINAATNLLP     120

PSEU2 MdtB      SSVTAPLERQFGQMPGLTQMASTSSGGASVITLRFSLEINMDVAEQQVQAAINAATNLLP     120

************************************************************

Translation TDLPAPPVYNKVNPADTPVLTLAITSKTMLLPRLNDLVDTRMAQKISQISGVGMVSIAGG     180

PSEU2 MdtB      TDLPAPPVYNKVNPADTPVLTLAITSKTMLLPKLNDLVDTRMAQKISQISGVGMVSIAGG     180

                ********************************:***************************

Translation QRQAVRIKVNPEALAANSLNLADVRTLISASNVNQPKGNFDGPTRVSMLDANDQLRSPEE     240

PSEU2 MdtB      QRQAVRIKVNPEALAANSLNLSDVRTLISASNVNQPKGNFDGPTRVSMLDANDQLKSPEE     240

                *********************:*********************************:****

Translation YANLILAYKDGAPLRLKDVAEIVDGAENERLAAWANRNQAVLLNIQRQPGANVIDVVDRI     300

PSEU2 MdtB      YANLILAYKDGAPLRLKDVAEIVNGAENERLAAWANRSQAVLLNIQRQPGANVIEVVDRI     300

                ***********************:*************.****************:*****

Translation KALLPGITDNLPAGLDVGGAD-PHADHSGVCHRCSARTADRHRAGGTGH-VPVSA-EVQR     357

PSEU2 MdtB      KALLPSITENLPAGLDVVVLTDRTQTIRASVTD-----VQHELLIAIVLVVLVTFLFLRR     355

                *****.**:********           .         .::.   .    * *:   ::*

Translation HNHS----LDCGTAVTGGHVRCHVPGRFLDQQPDLDGIDHRHRFRCRRRHRDAGKHLPAY     413

PSEU2 MdtB      FSATIIPSIAVPLSLVGTFGVMYLAG-FSVNNLTLMAMTIATGFVVDDAIVMLENISRHI     414

                .. :    :    ::.* .   :: * *  ::  * .:     *          :

Translation RRGRNPHAGRAQGRKTDRFHAD-LPDPVADCGADPA----AVHGRRGGA-----SVPRVR     463

PSEU2 MdtB      EEGETPLQAALKGAKQIGFTLISLTLSLIA-VLIPLLFMADVVGRLFREFAITLAVAILI     473

                ..*..*  .  :* *   *    *   :      *      * **         :*  :

Translation --DHPGGRHPDLAGGIADPDPDDVRAPAQTRAERGRPEPFLPGQ---WCVDRLADRYLC-     517

PSEU2 MdtB      SLVVSLTLTPMMCARLLKREPREEE---QSRF------YRASGAWIDWLIDIYAGRLRWV     524

                           * :.. : . :* : .   *:*           *    * :*  *.*

Translation ---GPVA----LGTQ----------ASAFDPAGRAGDAGVDCAAVYRGAQGLLSGAGHGS     560

PSEU2 MdtB      LKHQPLTLLVALATLALTVLLYIVVPKGFFPVQD---------------TGVIQGISEAP     569

                     *::    *.*            ..* *.                  *::.* ...

Translation DSRYFRST-----AVGFVRRHEPASTGT----------------RRHYSQGPGGGQPVVL     599

PSEU2 MdtB      QSVSFAAMSQRQQALADIILKDPAVVSLSSYIGVDGDNATLNSGRLLINLKPHGARDLTA     629

                 :*  * :      *:. :  ::** ..                 *   .  * *.: :.

Translation YWCR-WRQRNAQ------QWSPADQSQTA--PGTRPDRDRSHPAPATGGRQAFGHPPVHA     650

PSEU2 MdtB      SEVIQRLQPEVDKLSDIRLFMQPVQDLTIEDRVSRTQYQFSMSSPDAELLTLWSEKLVDA     689

                       * :.:       :    *. *     :* : : *  :* :     :..  *.*

Translation AGAGPDHRRPGQP-------YPVPVQHVIARR------RAVDALERKTGRRARQAG-TDR     696

PSEU2 MdtB      LGKRPELRDVASDLQDKGLQVYLNIDRDAASRVGVTVANITDALYDAFGQRQISTIYTQA     749

                 *  *: *  ..          : :::  * *      . .***    *:*  .:  *:

Translation CGQRPAGQGAAGVPEHRSRCG--QSGRRHGGQYHRCSV-RLRAAADFHHLHPGQPVPGGV     753

PSEU2 MdtB      SQYRVVLQAASGSELGPAALEQIHVKTTDGAQVKLSSLARVE------------------     791

                .  * . *.*:*     :     :    .*.* : .*: *:.

Translation AGCVRQRPGPGGAGADPRQDHRWRAGETVESGAHRAASGTVGHYSPGAV---------SG     804

PSEU2 MdtB      ------------------------------QRQAQLAIAHLGQFPAVMMSFNLAPDIALG     821

                                              .   : * . :*::    :          *

Translation GH-DVVQP-GARCCFGQGRAGDRAGPAGDWHADWRADAISGRC-----RSVSGVVVQHAA     857

PSEU2 MdtB      KAVKVIEEVEQEIGMPIGVQTQFQGAAEAFQASLSSTLLLILAAVVTMYIVLGVLYESYI     881

                   .*::    .  :  *   :  * *  ::*.  :  :   .       * **: :

Translation ADSGGSG-DHVHRAGRAL-ELHSPDHHPLDTAVGGCRCLAGVADQRQRSGHDRDHRHHSA     915

PSEU2 MdtB      HPITILSTLPSAAVGALLALLISGNDLGM-IAIIGIILLIGIVKKNAIMMIDFA---LDA     937

                                  .      .*  *  * * :.  :  *: *   * *:..:.     *      .*

Translation DRHRQ---EKRHHD---DRLRPGRRTKPRR-----------------RAGNGHL-SGAAA     951

PSEU2 MdtB      ERNRSVAPEQAIYDAALLRFRPILMTTLAALFGAIPLMLASGSGAELRQPLGLVMVGGLL     997

                            :*:*.   *:  :*    *:**   *.                    *   * :  *.

Translation LPANPDDHTGRVVRCFSADAGERLRCRIASAVGPGNGRRSVAEPSADAVYHAGHLPVLRS     1011

PSEU2 MdtB      LSQVLTLFTTPVIYLYFDRLGRRWSRKP---ADPDRQER------ADA------------     1036

                            *      .*  *:  :    *.*   :    ..*.. .*      ***

Translation SGQALEPQAGCPGSHRTG-RM     1031

PSEU2 MdtB      ---------------------     1036

Supplementary Figure 2. Sequence alignment of the translated DNA between the MdtA and MdtC in the P. aeruginosa strain lacking MdtB, and the MdtB from the most closely related P. aeruginosa (PSEU2).

# Loss of MdtABC in Some Organisms

Some organisms are in the process of losing one of the Mdt components. As an example, in Supplementary Figure 2 we show the translated DNA from between the *mdtA* and *mdtC* from *Pseudomonas syringae pv tomato* strain ATCC BAA-87, compared to the MdtB sequence from the most closely related *Pseudomonas syringae pv syringae* strain B728a. Up until amino acid 318 the sequences are practically identical, and afterwards they differ radically. Interestingly, the lost component was always an MdtB, suggesting the MdtC alone can retain some of the function, as suggested in the literature.

# Removed Sequences

Sequences that were removed prior to obtaining results shown in Figure 2 are: *Acinetobacter baylyi* Q6FD21, Q6FD22, Q6F786, Q6F787; *Gluconobacter oxydans* Q5FSC3; *Mesorhizobium japonicum* Q98B06; *Bradyrhizobium diazoefficiens* Q89XN1. They proved to be difficult to align to other RNDs, and thus their positions in the trees were highly sensitive to the methods used, and it is therefore possible these proteins are fusions of distantly related RNDs. Rerunning the analysis without these seven sequences significantly improved the bootstrap values of the tree, therefore we decided to exclude them from the main analysis.

*Acinetobacter baylyi* contains two sets of RND protein pairs, Q6FD21 & Q6FD22, and Q6F786 & Q6F787. Judging just by their genomic neighborhood they are both two RND subunit containing systems of similar architecture: MFP-RND1-RND2-OMP. The sequence identity and similarity values between the two systems in *A. baylyi* are low (52/69% and 40/57% for the corresponding B and C-like proteins), however, the Q6F786/Q6F787 system is most closely related to the MdtBC system from *Chromobacterium violaceum*, a Betaproteobacterium (67% and 63% sequence identity and 79% and 76% sequence similarity between the corresponding B and C-like proteins). This observation, together with the fact that no close homologues of the Q6F786/Q6F787 system were found in other *Acinetobacter* while it is abundant in *Chromobacteriaceae* suggests a horizontal gene transfer from *C. violaceum* or a close relative. The other pair, Q6FD21/Q6FD22, clustered together with other Gammaproteobacteria, however its actual placement was strongly sensitive to even the smallest variations in the sequence alignments.

The other two sequences (from *M. japonicum* and *B. diazoefficiens*) usually clustered together with “true” MexN-like proteins, yet with low bootstrap values and long branches. They are most likely results of a gene duplication of their respective “true” MexN-like proteins.

# Outer Membrane Proteins

It is particularly difficult to do phylogenetic analyses of outer membrane proteins due to their unique architecture: the outwards facing transmembrane residues are never strongly conserved, seriously decreasing the number of residues available for alignment. Moreover, most outer membrane proteins have similar 3D structure despite high divergence in sequences. Nevertheless, our analysis of the outer membrane factors from the Mdt/Mex systems supports the evolutionary scenario proposed in Figure 3. As shown in Supplementary Figure 3, the OMFs of the “progenitor-like” RNDs separate first from the rest, followed by Alpha OMFs. The rest was Beta and Gammaproteobacteria, and the branching follows the one seen in Figure 2.


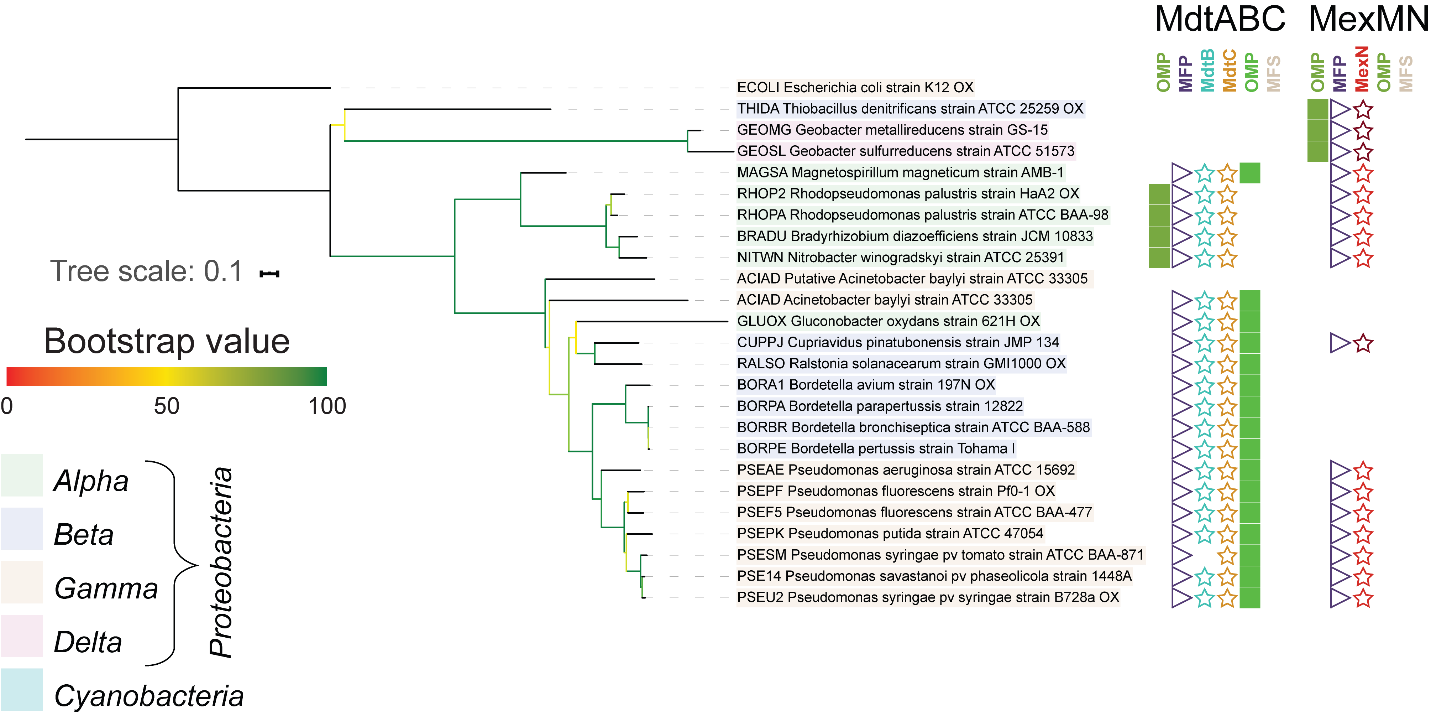


Supplementary Figure 3. A phylogenetic tree of outer membrane factors from the Mdt/Mex systems, rooted on distantly related TolC from E. coli.

# Membrane Fusion Proteins

As a complement to the analysis of the RND proteins, their corresponding MFPs were aligned, and phylogeny reconstructed (Supplementary Figure 4). However, due to their shorter lengths and lower conservation levels the results were less reliable and therefore excluded from the main text. Nevertheless, the general conclusion is similar to the one obtained by comparing RNDs: the “progenitor-like” RNDs separate earliest from the rest.


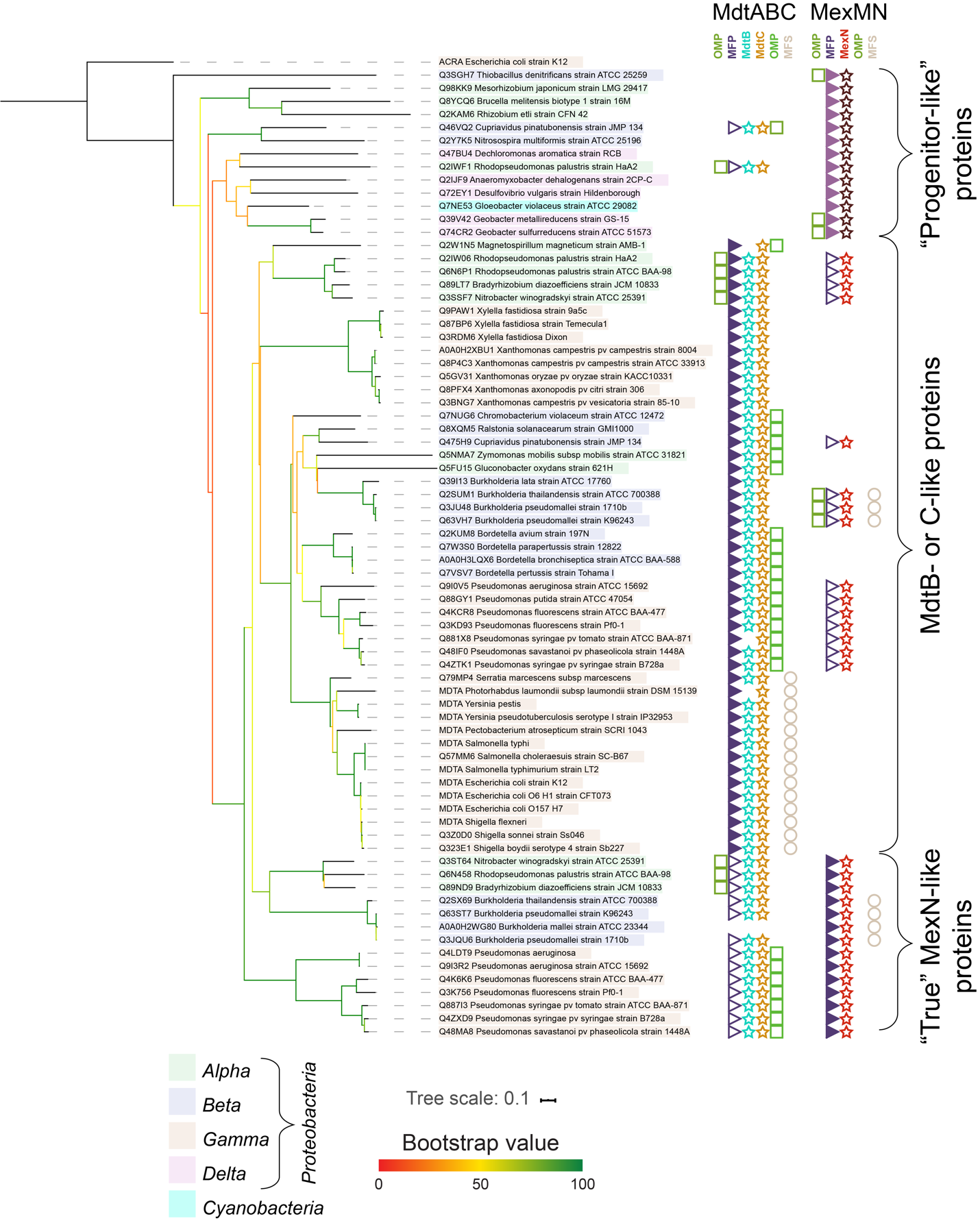


Supplementary Figure 4. A phylogenetic tree of membrane fusion proteins, rooted on distantly related E. coli AcrA. The annotations are the same as in Fig. 2.

# Sequences used for the analysis

| **Name** | **Organism** | **Description** |
| --- | --- | --- |
| A0A0F7RLT4 | Bacillus anthracis. | Transporter\| AcrB/AcrD/AcrF family |
| A0A0H2UYQ5 | Shigella flexneri. | Efflux pump membrane transporter |
| A0A0H2V123 | Shigella flexneri. | Efflux pump membrane transporter |
| A0A0H2V4W9 | Escherichia coli O6:H1 (strain CFT073 / ATCC 700928 / UPEC) | Efflux pump membrane transporter |
| A0A0H2V703 | Escherichia coli O6:H1 (strain CFT073 / ATCC 700928 / UPEC) | Efflux pump membrane transporter |
| A0A0H2V9F8 | Escherichia coli O6:H1 (strain CFT073 / ATCC 700928 / UPEC) | Efflux pump membrane transporter |
| A0A0H2VBM4 | Escherichia coli O6:H1 (strain CFT073 / ATCC 700928 / UPEC) | Efflux pump membrane transporter |
| A0A0H2VIZ4 | Staphylococcus epidermidis (strain ATCC 12228) | Acriflavin resistance protein |
| A0A0H2W6E0 | Yersinia pestis. | Efflux pump membrane transporter |
| A0A0H2WBD9 | Burkholderia mallei (strain ATCC 23344) | Heavy metal efflux pump CzcA |
| A0A0H2WCV6 | Burkholderia mallei (strain ATCC 23344) | Hydrophobe/amphiphile efflux family protein |
| A0A0H2WD52 | Burkholderia mallei (strain ATCC 23344) | Efflux pump membrane transporter |
| A0A0H2WG76 | Burkholderia mallei (strain ATCC 23344) | AcrB/AcrD/AcrF family protein |
| A0A0H2WHE9 | Burkholderia mallei (strain ATCC 23344) | Heavy metal efflux pump\| CzcA family |
| A0A0H2WHU3 | Burkholderia mallei (strain ATCC 23344) | Efflux pump membrane transporter |
| A0A0H2WIU9 | Burkholderia mallei (strain ATCC 23344) | AcrB/AcrD/AcrF family protein |
| A0A0H2WN05 | Salmonella paratyphi A (strain ATCC 9150 / SARB42) | Efflux pump membrane transporter |
| A0A0H2WS58 | Salmonella paratyphi A (strain ATCC 9150 / SARB42) | Efflux pump membrane transporter |
| A0A0H2WTM3 | Salmonella paratyphi A (strain ATCC 9150 / SARB42) | Efflux pump membrane transporter |
| A0A0H2WWS3 | Staphylococcus aureus (strain COL) | AcrB/AcrD/AcrF family protein |
| A0A0H2X3D6 | Xanthomonas campestris pv. campestris (strain 8004) | Cation efflux system protein |
| A0A0H2X4R2 | Xanthomonas campestris pv. campestris (strain 8004) | Acriflavin resistance protein |
| A0A0H2X699 | Xanthomonas campestris pv. campestris (strain 8004) | Efflux pump membrane transporter |
| A0A0H2X729 | Xanthomonas campestris pv. campestris (strain 8004) | Transport protein |
| A0A0H2X7G8 | Xanthomonas campestris pv. campestris (strain 8004) | Efflux pump membrane transporter |
| A0A0H2X8U8 | Xanthomonas campestris pv. campestris (strain 8004) | Transport protein |
| A0A0H2XAS3 | Xanthomonas campestris pv. campestris (strain 8004) | Efflux pump membrane transporter |
| A0A0H2XBS4 | Xanthomonas campestris pv. campestris (strain 8004) | Acriflavin resistance protein |
| A0A0H2XE02 | Xanthomonas campestris pv. campestris (strain 8004) | Cation efflux system protein |
| A0A0H3G1R6 | Brucella suis biovar 1 (strain 1330) | AcrB/AcrD/AcrF multidrug efflux protein |
| A0A0H3G714 | Brucella suis biovar 1 (strain 1330) | AcrB/AcrD/AcrF multidrug efflux protein |
| A0A0H3GFZ4 | Brucella suis biovar 1 (strain 1330) | AcrB/AcrD/AcrF multidrug efflux protein |
| A0A0H3JMN8 | Escherichia coli O157:H7. | Efflux pump membrane transporter |
| A0A0H3JN48 | Staphylococcus aureus (strain N315) | SA2056 protein |
| A0A0H3K0N2 | Staphylococcus aureus (strain Mu50 / ATCC 700699) | Similar to acriflavin resistance protein |
| A0A0H3K3Z1 | Synechococcus sp. (strain ATCC 27144 / PCC 6301 / SAUG 1402/1) | RND multidrug efflux transporter |
| A0A0H3K464 | Staphylococcus aureus (strain MW2) | MW2179 protein |
| A0A0H3K4S1 | Synechococcus sp. (strain ATCC 27144 / PCC 6301 / SAUG 1402/1) | Probable cation efflux system protein |
| A0A0H3LLX7 | Bordetella bronchiseptica (strain ATCC BAA-588 / NCTC 13252 / RB50) | Efflux pump membrane transporter |
| A0A0H3LQU0 | Bordetella bronchiseptica (strain ATCC BAA-588 / NCTC 13252 / RB50) | Efflux pump membrane transporter |
| A0A0H3LQV1 | Bordetella bronchiseptica (strain ATCC BAA-588 / NCTC 13252 / RB50) | AcrB/AcrD/AcrF family protein |
| A0A0H3LT18 | Bordetella bronchiseptica (strain ATCC BAA-588 / NCTC 13252 / RB50) | Efflux pump membrane transporter |
| A0A0H3LT79 | Bartonella quintana (strain Toulouse) | Acriflavin resistance protein d |
| A0A0H3LUJ4 | Bordetella bronchiseptica (strain ATCC BAA-588 / NCTC 13252 / RB50) | Integral membrane component of multidrug efflux system |
| A0A0H3LWT2 | Bordetella bronchiseptica (strain ATCC BAA-588 / NCTC 13252 / RB50) | Probable membrane protein |
| A0A0H3LZH6 | Bordetella bronchiseptica (strain ATCC BAA-588 / NCTC 13252 / RB50) | AcrB/AcrD/AcrF family protein |
| A0A0H3M8G5 | Ehrlichia ruminantium (strain Welgevonden) | Probable aminoglycoside efflux pump (Acriflavine resistance protein D) |
| A0A0R4J7B3 | Bartonella henselae (strain ATCC 49882 / DSM 28221 / Houston 1) | Acriflavin resistance protein d |
| O06471 | Burkholderia cepacia | Efflux pump membrane transporter |
| O25328 | Helicobacter pylori (strain ATCC 700392 / 26695) | Acriflavine resistance protein (AcrB) |
| O25622 | Helicobacter pylori (strain ATCC 700392 / 26695) | Cation efflux system protein (CzcA) |
| O25887 | Helicobacter pylori (strain ATCC 700392 / 26695) | Cation efflux system protein (CzcA) |
| O31100 | Pseudomonas putida | Solvent-resistant pump membrane transporter SrpB |
| O31501 | Bacillus subtilis (strain 168) | Swarming motility protein SwrC |
| O52248 | Pseudomonas putida (strain DOT-T1E) | Toluene efflux pump membrane transporter TtgB |
| O66770 | Aquifex aeolicus (strain VF5) | Cation efflux system (AcrB/AcrD/AcrF family) |
| O66916 | Aquifex aeolicus (strain VF5) | Cation efflux system (AcrB/AcrD/AcrF family) |
| O66977 | Aquifex aeolicus (strain VF5) | Cation efflux (AcrB/AcrD/AcrF family) |
| O68962 | Helicobacter pylori | RND pump protein |
| O87936 | Burkholderia pseudomallei | Efflux pump membrane transporter |
| P0C070 | Pseudomonas putida | Multidrug/solvent efflux pump membrane transporter MepB |
| P13511 | Cupriavidus metallidurans (strain ATCC 43123 / DSM 2839 / NBRC 102507/ CH34) | Cobalt-zinc-cadmium resistance protein CzcA |
| P24177 | Escherichia coli (strain K12) | Probable aminoglycoside efflux pump |
| P24181 | Escherichia coli (strain K12) | Multidrug export protein AcrF |
| P25197 | Rhizobium meliloti (strain 1021) | Nodulation protein NolG |
| P31224 | Escherichia coli (strain K12) | Multidrug efflux pump subunit AcrB |
| P37637 | Escherichia coli (strain K12) | Multidrug resistance protein MdtF |
| P37972 | Cupriavidus metallidurans (strain ATCC 43123 / DSM 2839 / NBRC 102507/ CH34) | Nickel and cobalt resistance protein CnrA |
| P38054 | Escherichia coli (strain K12) | Cation efflux system protein CusA |
| P52002 | Pseudomonas aeruginosa (strain ATCC 15692 / DSM 22644 / CIP 104116 /JCM 14847 / LMG 12228 / 1C / PRS 101 / PAO1) | Multidrug resistance protein MexB |
| P69340 | Shigella flexneri. | Multidrug resistance protein MdtB |
| P73998 | Synechocystis sp. (strain PCC 6803 / Kazusa) | Cation or drug efflux system protein |
| P74461 | Synechocystis sp. (strain PCC 6803 / Kazusa) | Cation or drug efflux system protein |
| P76398 | Escherichia coli (strain K12) | Multidrug resistance protein MdtB |
| P76399 | Escherichia coli (strain K12) | Multidrug resistance protein MdtC |
| P94177 | Alcaligenes sp. (strain CT14) | Cation efflux system protein CzcA |
| P95422 | Pseudomonas aeruginosa. | Efflux pump membrane transporter |
| Q2IGB6 | Anaeromyxobacter dehalogenans (strain 2CP-C) | Heavy metal efflux pump CzcA |
| Q2IGC8 | Anaeromyxobacter dehalogenans (strain 2CP-C) | Acriflavin resistance protein |
| Q2IGK2 | Anaeromyxobacter dehalogenans (strain 2CP-C) | Acriflavin resistance protein |
| Q2IHW6 | Anaeromyxobacter dehalogenans (strain 2CP-C) | Acriflavin resistance protein |
| Q2IJG5 | Anaeromyxobacter dehalogenans (strain 2CP-C) | Acriflavin resistance protein |
| Q2IK25 | Anaeromyxobacter dehalogenans (strain 2CP-C) | Acriflavin resistance protein |
| Q2IMH0 | Anaeromyxobacter dehalogenans (strain 2CP-C) | Acriflavin resistance protein |
| Q2INB4 | Anaeromyxobacter dehalogenans (strain 2CP-C) | Efflux pump membrane transporter |
| Q2IPR5 | Anaeromyxobacter dehalogenans (strain 2CP-C) | Heavy metal efflux pump CzcA |
| Q2IQ78 | Anaeromyxobacter dehalogenans (strain 2CP-C) | Acriflavin resistance protein |
| Q2IS23 | Rhodopseudomonas palustris (strain HaA2) | Acriflavin resistance protein |
| Q2IS29 | Rhodopseudomonas palustris (strain HaA2) | Heavy metal efflux pump CzcA |
| Q2IS98 | Rhodopseudomonas palustris (strain HaA2) | Acriflavin resistance protein |
| Q2ISU1 | Rhodopseudomonas palustris (strain HaA2) | Efflux pump membrane transporter |
| Q2ITW6 | Rhodopseudomonas palustris (strain HaA2) | Acriflavin resistance protein |
| Q2IU06 | Rhodopseudomonas palustris (strain HaA2) | Acriflavin resistance protein |
| Q2IU55 | Rhodopseudomonas palustris (strain HaA2) | Heavy metal efflux pump CzcA |
| Q2IUK5 | Rhodopseudomonas palustris (strain HaA2) | Acriflavin resistance protein |
| Q2IUW1 | Rhodopseudomonas palustris (strain HaA2) | Efflux pump membrane transporter |
| Q2IW07 | Rhodopseudomonas palustris (strain HaA2) | Acriflavin resistance protein |
| Q2IW08 | Rhodopseudomonas palustris (strain HaA2) | Acriflavin resistance protein |
| Q2IWF2 | Rhodopseudomonas palustris (strain HaA2) | Acriflavin resistance protein |
| Q2IWZ0 | Rhodopseudomonas palustris (strain HaA2) | Efflux pump membrane transporter |
| Q2IXW6 | Rhodopseudomonas palustris (strain HaA2) | Efflux pump membrane transporter |
| Q2J147 | Rhodopseudomonas palustris (strain HaA2) | Heavy metal efflux pump CzcA |
| Q2JZ12 | Rhizobium etli (strain CFN 42 / ATCC 51251) | Probable efflux transporter protein\| acriflavin resistance protein family |
| Q2K1A4 | Rhizobium etli (strain CFN 42 / ATCC 51251) | Probable efflux cation transporter protein |
| Q2K3Z4 | Rhizobium etli (strain CFN 42 / ATCC 51251) | Putative multidrug efflux transporter protein |
| Q2K4R7 | Rhizobium etli (strain CFN 42 / ATCC 51251) | Efflux pump membrane transporter |
| Q2K4W2 | Rhizobium etli (strain CFN 42 / ATCC 51251) | Efflux pump membrane transporter |
| Q2K4X5 | Rhizobium etli (strain CFN 42 / ATCC 51251) | Efflux pump membrane transporter |
| Q2K6F6 | Rhizobium etli (strain CFN 42 / ATCC 51251) | Probable acriflavin resistance (Multidrug efflux transporter) protein |
| Q2K7A2 | Rhizobium etli (strain CFN 42 / ATCC 51251) | Probable cation efflux system protein (Heavy metal efflux pump)\| CzcA family |
| Q2KAM7 | Rhizobium etli (strain CFN 42 / ATCC 51251) | Probable multidrug efflux transporter protein\| AcrB/AcrD/AcrF family |
| Q2KCU0 | Rhizobium etli (strain CFN 42 / ATCC 51251) | Probable cation/multidrug efflux transport protein |
| Q2KUN1 | Bordetella avium (strain 197N) | Multidrug efflux system transmembrane protein |
| Q2KUN3 | Bordetella avium (strain 197N) | Multidrug efflux system transmembrane protein |
| Q2KV59 | Bordetella avium (strain 197N) | Multidrug efflux system transmembrane protein |
| Q2KXM0 | Bordetella avium (strain 197N) | Efflux pump membrane transporter |
| Q2KZR5 | Bordetella avium (strain 197N) | Efflux pump membrane transporter |
| Q2L1H0 | Bordetella avium (strain 197N) | Efflux pump membrane transporter |
| Q2LRD7 | Syntrophus aciditrophicus (strain SB) | Efflux pump membrane transporter |
| Q2LY61 | Syntrophus aciditrophicus (strain SB) | Efflux pump membrane transporter |
| Q2NBE8 | Erythrobacter litoralis (strain HTCC2594) | Heavy metal RND efflux transporter\| CzcA family protein |
| Q2NBG2 | Erythrobacter litoralis (strain HTCC2594) | Metal ion efflux RND protein family protein |
| Q2NV66 | Sodalis glossinidius (strain morsitans) | Efflux pump membrane transporter |
| Q2RFY8 | Moorella thermoacetica (strain ATCC 39073 / JCM 9320) | Acriflavin resistance protein |
| Q2RN02 | Rhodospirillum rubrum (strain ATCC 11170 / ATH 1.1.1 / DSM 467 / LMG4362 / NCIB 8255 / S1) | Acriflavin resistance protein |
| Q2RQ71 | Rhodospirillum rubrum (strain ATCC 11170 / ATH 1.1.1 / DSM 467 / LMG4362 / NCIB 8255 / S1) | Acriflavin resistance protein |
| Q2RSK1 | Rhodospirillum rubrum (strain ATCC 11170 / ATH 1.1.1 / DSM 467 / LMG4362 / NCIB 8255 / S1) | Acriflavin resistance protein |
| Q2RSP4 | Rhodospirillum rubrum (strain ATCC 11170 / ATH 1.1.1 / DSM 467 / LMG4362 / NCIB 8255 / S1) | Efflux pump membrane transporter |
| Q2RVC6 | Rhodospirillum rubrum (strain ATCC 11170 / ATH 1.1.1 / DSM 467 / LMG4362 / NCIB 8255 / S1) | Efflux pump membrane transporter |
| Q2RW08 | Rhodospirillum rubrum (strain ATCC 11170 / ATH 1.1.1 / DSM 467 / LMG4362 / NCIB 8255 / S1) | Efflux pump membrane transporter |
| Q2RYR4 | Salinibacter ruber (strain DSM 13855 / M31) | Cation efflux system protein czcA |
| Q2S141 | Salinibacter ruber (strain DSM 13855 / M31) | Transporter\| AcrB/D/F family |
| Q2S3C4 | Salinibacter ruber (strain DSM 13855 / M31) | Acriflavine resistance protein (Cation efflux system) |
| Q2S4L8 | Salinibacter ruber (strain DSM 13855 / M31) | Multidrug resistance protein\| putative |
| Q2S5B3 | Salinibacter ruber (strain DSM 13855 / M31) | Multidrug efflux transporter\| AcrB/AcrD/AcrF family |
| Q2S5Q5 | Salinibacter ruber (strain DSM 13855 / M31) | Cation/multidrug efflux pump |
| Q2S6E3 | Salinibacter ruber (strain DSM 13855 / M31) | Transporter\| AcrB/D/F family |
| Q2S716 | Hahella chejuensis (strain KCTC 2396) | Efflux pump membrane transporter |
| Q2S7Z8 | Hahella chejuensis (strain KCTC 2396) | Cation/multidrug efflux pump |
| Q2SB32 | Hahella chejuensis (strain KCTC 2396) | Cation/multidrug efflux pump |
| Q2SET3 | Hahella chejuensis (strain KCTC 2396) | Cation/multidrug efflux pump |
| Q2SF27 | Hahella chejuensis (strain KCTC 2396) | Cation/multidrug efflux pump |
| Q2SFQ4 | Hahella chejuensis (strain KCTC 2396) | Efflux pump membrane transporter |
| Q2SFT2 | Hahella chejuensis (strain KCTC 2396) | Cation/multidrug efflux pump |
| Q2SHT7 | Hahella chejuensis (strain KCTC 2396) | Putative silver efflux pump |
| Q2SJY0 | Hahella chejuensis (strain KCTC 2396) | Cation/multidrug efflux pump |
| Q2SM00 | Hahella chejuensis (strain KCTC 2396) | Cation/multidrug efflux pump |
| Q2SUM0 | Burkholderia thailandensis (strain ATCC 700388 / DSM 13276 / CIP106301 / E264) | AcrB/AcrD/AcrF family protein |
| Q2SVT4 | Burkholderia thailandensis (strain ATCC 700388 / DSM 13276 / CIP106301 / E264) | Efflux pump membrane transporter |
| Q2SW90 | Burkholderia thailandensis (strain ATCC 700388 / DSM 13276 / CIP106301 / E264) | AcrB/AcrD/AcrF family protein |
| Q2SX70 | Burkholderia thailandensis (strain ATCC 700388 / DSM 13276 / CIP106301 / E264) | AcrB/AcrD/AcrF family protein |
| Q2T0R2 | Burkholderia thailandensis (strain ATCC 700388 / DSM 13276 / CIP106301 / E264) | Efflux pump membrane transporter |
| Q2T3F6 | Burkholderia thailandensis (strain ATCC 700388 / DSM 13276 / CIP106301 / E264) | Efflux pump membrane transporter |
| Q2T5H8 | Burkholderia thailandensis (strain ATCC 700388 / DSM 13276 / CIP106301 / E264) | Heavy metal efflux pump CzcA |
| Q2T5R6 | Burkholderia thailandensis (strain ATCC 700388 / DSM 13276 / CIP106301 / E264) | Hydrophobe/amphiphile efflux family protein |
| Q2T618 | Burkholderia thailandensis (strain ATCC 700388 / DSM 13276 / CIP106301 / E264) | Heavy metal efflux pump CzcA\| putative |
| Q2T989 | Burkholderia thailandensis (strain ATCC 700388 / DSM 13276 / CIP106301 / E264) | Transporter\| AcrB/D/F family |
| Q2W1N8 | Magnetospirillum magneticum (strain AMB-1 / ATCC 700264) | Cation/multidrug efflux pump |
| Q2W1P6 | Magnetospirillum magneticum (strain AMB-1 / ATCC 700264) | Efflux pump membrane transporter |
| Q2W1Q9 | Magnetospirillum magneticum (strain AMB-1 / ATCC 700264) | Cation/multidrug efflux pump |
| Q2W646 | Magnetospirillum magneticum (strain AMB-1 / ATCC 700264) | Efflux pump membrane transporter |
| Q2W7X3 | Magnetospirillum magneticum (strain AMB-1 / ATCC 700264) | Putative silver efflux pump |
| Q2Y770 | Nitrosospira multiformis (strain ATCC 25196 / NCIMB 11849 / C 71) | Cobalt-zinc-cadmium resistance protein CzcA |
| Q2Y7K6 | Nitrosospira multiformis (strain ATCC 25196 / NCIMB 11849 / C 71) | Acriflavin resistance protein |
| Q2Y896 | Nitrosospira multiformis (strain ATCC 25196 / NCIMB 11849 / C 71) | Efflux pump membrane transporter |
| Q2Y8I2 | Nitrosospira multiformis (strain ATCC 25196 / NCIMB 11849 / C 71) | Cobalt-zinc-cadmium resistance protein CzcA |
| Q2Y962 | Nitrosospira multiformis (strain ATCC 25196 / NCIMB 11849 / C 71) | Cu(I)/Ag(I) efflux system membrane protein CusA/SilA |
| Q2YA05 | Nitrosospira multiformis (strain ATCC 25196 / NCIMB 11849 / C 71) | Cobalt-zinc-cadmium resistance protein CzcA |
| Q2YAR7 | Nitrosospira multiformis (strain ATCC 25196 / NCIMB 11849 / C 71) | Efflux pump membrane transporter |
| Q2YB73 | Nitrosospira multiformis (strain ATCC 25196 / NCIMB 11849 / C 71) | Efflux pump membrane transporter |
| Q2YB76 | Nitrosospira multiformis (strain ATCC 25196 / NCIMB 11849 / C 71) | Efflux pump membrane transporter |
| Q2YD19 | Nitrosospira multiformis (strain ATCC 25196 / NCIMB 11849 / C 71) | Efflux pump membrane transporter |
| Q2YIU1 | Brucella abortus (strain 2308) | Acriflavin resistance protein |
| Q2YPC8 | Brucella abortus (strain 2308) | Acriflavin resistance protein |
| Q2YPE6 | Brucella abortus (strain 2308) | Efflux pump membrane transporter |
| Q2YPZ1 | Brucella abortus (strain 2308) | Acriflavin resistance protein |
| Q2Z053 | uncultured bacterium. | Heavy metal efflux pump |
| Q30B59 | Acinetobacter sp. 4365. | Efflux pump membrane transporter |
| Q30NZ6 | Sulfurimonas denitrificans (strain ATCC 33889 / DSM 1251) | Resistance-Nodulation-Cell Division Superfamily transporter |
| Q30QL4 | Sulfurimonas denitrificans (strain ATCC 33889 / DSM 1251) | Resistance-Nodulation-Cell Division Superfamily transporter |
| Q30S70 | Sulfurimonas denitrificans (strain ATCC 33889 / DSM 1251) | Resistance-Nodulation-Cell Division Superfamily transporter |
| Q30T66 | Sulfurimonas denitrificans (strain ATCC 33889 / DSM 1251) | Efflux pump membrane transporter |
| Q311N6 | Desulfovibrio alaskensis (strain G20) | Acriflavin resistance protein |
| Q313Y0 | Desulfovibrio alaskensis (strain G20) | Acriflavin resistance protein |
| Q315C5 | Desulfovibrio alaskensis (strain G20) | Acriflavin resistance protein |
| Q315P7 | Desulfovibrio alaskensis (strain G20) | Acriflavin resistance protein |
| Q316E4 | Desulfovibrio alaskensis (strain G20) | Efflux pump membrane transporter |
| Q31DT9 | Hydrogenovibrio crunogenus (strain XCL-2) | Resistance-Nodulation-Cell Division (RND) superfamily cation efflux transporter |
| Q31E33 | Hydrogenovibrio crunogenus (strain XCL-2) | Resistance-Nodulation-Cell Division (RND) superfamily cation efflux transporter |
| Q31EH5 | Hydrogenovibrio crunogenus (strain XCL-2) | Resistance-Nodulation-Cell Division (RND) superfamily cation efflux transporter |
| Q31EQ3 | Hydrogenovibrio crunogenus (strain XCL-2) | Resistance-Nodulation-Cell Division (RND) superfamily cation efflux transporter |
| Q31EX8 | Hydrogenovibrio crunogenus (strain XCL-2) | Resistance-Nodulation-Cell Division (RND) superfamily cation efflux transporter |
| Q31FE9 | Hydrogenovibrio crunogenus (strain XCL-2) | Efflux pump membrane transporter |
| Q31FQ2 | Hydrogenovibrio crunogenus (strain XCL-2) | Resistance-Nodulation-Cell Division (RND) superfamily cation efflux transporter |
| Q31J34 | Hydrogenovibrio crunogenus (strain XCL-2) | Resistance-Nodulation-Cell Division (RND) superfamily cation efflux transporter |
| Q31JL9 | Hydrogenovibrio crunogenus (strain XCL-2) | Resistance-Nodulation-Cell Division (RND) superfamily transporter |
| Q31JR8 | Hydrogenovibrio crunogenus (strain XCL-2) | Resistance-Nodulation-Cell Division (RND) superfamily cation efflux transporter |
| Q31KM0 | Synechococcus elongatus (strain PCC 7942) | Hydrophobe/amphiphile efflux-1 HAE1 |
| Q31M20 | Synechococcus elongatus (strain PCC 7942) | Probable cation efflux system protein |
| Q31VB1 | Shigella boydii serotype 4 (strain Sb227) | Efflux pump membrane transporter |
| Q31W02 | Shigella boydii serotype 4 (strain Sb227) | Efflux pump membrane transporter |
| Q323D9 | Shigella boydii serotype 4 (strain Sb227) | Multidrug resistance protein MdtC |
| Q323E0 | Shigella boydii serotype 4 (strain Sb227) | Multidrug resistance protein MdtB |
| Q324W8 | Shigella boydii serotype 4 (strain Sb227) | Putative inner membrane component for iron transport |
| Q325D4 | Shigella boydii serotype 4 (strain Sb227) | Efflux pump membrane transporter |
| Q32AZ2 | Shigella dysenteriae serotype 1 (strain Sd197) | Efflux pump membrane transporter |
| Q32DA0 | Shigella dysenteriae serotype 1 (strain Sd197) | Efflux pump membrane transporter |
| Q32GN8 | Shigella dysenteriae serotype 1 (strain Sd197) | Efflux pump membrane transporter |
| Q32J42 | Shigella dysenteriae serotype 1 (strain Sd197) | Efflux pump membrane transporter |
| Q392S4 | Burkholderia lata (strain ATCC 17760 / DSM 23089 / LMG 22485 / NCIMB9086 / R18194 / 383) | Heavy metal efflux pump\| CzcA family |
| Q395M1 | Burkholderia lata (strain ATCC 17760 / DSM 23089 / LMG 22485 / NCIMB9086 / R18194 / 383) | Acriflavin resistance protein |
| Q397Q3 | Burkholderia lata (strain ATCC 17760 / DSM 23089 / LMG 22485 / NCIMB9086 / R18194 / 383) | Efflux pump membrane transporter |
| Q398J2 | Burkholderia lata (strain ATCC 17760 / DSM 23089 / LMG 22485 / NCIMB9086 / R18194 / 383) | Efflux pump membrane transporter |
| Q39AU9 | Burkholderia lata (strain ATCC 17760 / DSM 23089 / LMG 22485 / NCIMB9086 / R18194 / 383) | Efflux pump membrane transporter |
| Q39DD3 | Burkholderia lata (strain ATCC 17760 / DSM 23089 / LMG 22485 / NCIMB9086 / R18194 / 383) | Efflux pump membrane transporter |
| Q39FY6 | Burkholderia lata (strain ATCC 17760 / DSM 23089 / LMG 22485 / NCIMB9086 / R18194 / 383) | Acriflavin resistance protein |
| Q39G27 | Burkholderia lata (strain ATCC 17760 / DSM 23089 / LMG 22485 / NCIMB9086 / R18194 / 383) | Efflux pump membrane transporter |
| Q39GQ3 | Burkholderia lata (strain ATCC 17760 / DSM 23089 / LMG 22485 / NCIMB9086 / R18194 / 383) | Efflux pump membrane transporter |
| Q39I14 | Burkholderia lata (strain ATCC 17760 / DSM 23089 / LMG 22485 / NCIMB9086 / R18194 / 383) | Acriflavin resistance protein |
| Q39NU6 | Burkholderia lata (strain ATCC 17760 / DSM 23089 / LMG 22485 / NCIMB9086 / R18194 / 383) | Efflux pump membrane transporter |
| Q39PF8 | Burkholderia lata (strain ATCC 17760 / DSM 23089 / LMG 22485 / NCIMB9086 / R18194 / 383) | Efflux pump membrane transporter |
| Q39PW0 | Geobacter metallireducens (strain GS-15 / ATCC 53774 / DSM 7210) | Efflux pump\| RND family\| inner membrane protein |
| Q39QY3 | Geobacter metallireducens (strain GS-15 / ATCC 53774 / DSM 7210) | Efflux pump\| RND family\| inner membrane protein |
| Q39SN5 | Geobacter metallireducens (strain GS-15 / ATCC 53774 / DSM 7210) | Efflux pump\| RND family\| inner membrane protein |
| Q39V29 | Geobacter metallireducens (strain GS-15 / ATCC 53774 / DSM 7210) | Efflux pump membrane transporter |
| Q39V41 | Geobacter metallireducens (strain GS-15 / ATCC 53774 / DSM 7210) | Efflux pump\| RND family\| inner membrane protein\| AcrB/AcrD/AcrF family |
| Q39VE3 | Geobacter metallireducens (strain GS-15 / ATCC 53774 / DSM 7210) | Metal ion efflux pump\| RND family\| inner membrane protein |
| Q39XH2 | Geobacter metallireducens (strain GS-15 / ATCC 53774 / DSM 7210) | Efflux pump membrane transporter |
| Q39ZX7 | Pelobacter carbinolicus (strain DSM 2380 / NBRC 103641 / GraBd1) | Efflux pump membrane transporter |
| Q3A2C4 | Pelobacter carbinolicus (strain DSM 2380 / NBRC 103641 / GraBd1) | Efflux pump\| RND family\| inner membrane protein\| AcrB/AcrD/AcrF family |
| Q3A5K8 | Pelobacter carbinolicus (strain DSM 2380 / NBRC 103641 / GraBd1) | Efflux pump membrane transporter |
| Q3A6S9 | Pelobacter carbinolicus (strain DSM 2380 / NBRC 103641 / GraBd1) | Efflux pump\| RND family\| inner membrane protein |
| Q3A7U6 | Pelobacter carbinolicus (strain DSM 2380 / NBRC 103641 / GraBd1) | Efflux pump membrane transporter |
| Q3ARB0 | Chlorobium chlorochromatii (strain CaD3) | Hydrophobe/amphiphile efflux-1 HAE1 |
| Q3ARZ1 | Chlorobium chlorochromatii (strain CaD3) | NolG efflux transporter |
| Q3AUC4 | Chlorobium chlorochromatii (strain CaD3) | AcrB/AcrD/AcrF family protein |
| Q3B1E0 | Chlorobium luteolum (strain DSM 273 / 2530) | AcrB/AcrD/AcrF family protein |
| Q3B4Q3 | Chlorobium luteolum (strain DSM 273 / 2530) | Hydrophobe/amphiphile efflux-1 HAE1 |
| Q3B4Z4 | Chlorobium luteolum (strain DSM 273 / 2530) | RND family efflux transporter |
| Q3BMM3 | Xanthomonas campestris pv. vesicatoria (strain 85-10) | RND superfamily protein |
| Q3BNG5 | Xanthomonas campestris pv. vesicatoria (strain 85-10) | RND superfamily protein |
| Q3BPY9 | Xanthomonas campestris pv. vesicatoria (strain 85-10) | RND superfamily protein |
| Q3BR79 | Xanthomonas campestris pv. vesicatoria (strain 85-10) | Efflux pump membrane transporter |
| Q3BRC2 | Xanthomonas campestris pv. vesicatoria (strain 85-10) | RND superfamily protein |
| Q3BS55 | Xanthomonas campestris pv. vesicatoria (strain 85-10) | Efflux pump membrane transporter |
| Q3BSU9 | Xanthomonas campestris pv. vesicatoria (strain 85-10) | Efflux pump membrane transporter |
| Q3BTF0 | Xanthomonas campestris pv. vesicatoria (strain 85-10) | RND superfamily protein |
| Q3BTF1 | Xanthomonas campestris pv. vesicatoria (strain 85-10) | RND superfamily protein |
| Q3BTS5 | Xanthomonas campestris pv. vesicatoria (strain 85-10) | RND superfamily protein |
| Q3BVE1 | Xanthomonas campestris pv. vesicatoria (strain 85-10) | Efflux pump membrane transporter |
| Q3BYG6 | Xanthomonas campestris pv. vesicatoria (strain 85-10) | RND superfamily protein |
| Q3BYU5 | Xanthomonas campestris pv. vesicatoria (strain 85-10) | Efflux pump membrane transporter |
| Q3ER77 | Bacillus thuringiensis serovar israelensis ATCC 35646. | Acriflavin resistance plasma membrane protein |
| Q3IBZ0 | Pseudoalteromonas haloplanktis (strain TAC 125) | Efflux pump membrane transporter |
| Q3IC20 | Pseudoalteromonas haloplanktis (strain TAC 125) | Efflux pump membrane transporter |
| Q3ICA5 | Pseudoalteromonas haloplanktis (strain TAC 125) | Putative metabolite exporter\| AcrB/D/F family |
| Q3ICE4 | Pseudoalteromonas haloplanktis (strain TAC 125) | Putative metabolite exporter\| AcrB/D/F family |
| Q3IHS9 | Pseudoalteromonas haloplanktis (strain TAC 125) | Putative acrB/acrD/acrF acriflavin resistance family protein |
| Q3IK01 | Pseudoalteromonas haloplanktis (strain TAC 125) | Putative multidrug resistance protein\| AcrB/AcrD/AcrF family |
| Q3IKS0 | Pseudoalteromonas haloplanktis (strain TAC 125) | Putative transport protein |
| Q3ILD1 | Pseudoalteromonas haloplanktis (strain TAC 125) | Cation efflux system protein cusA |
| Q3ILG7 | Pseudoalteromonas haloplanktis (strain TAC 125) | Cobalt-zinc-cadmium resistance protein czcA (Cation efflux system protein czcA) |
| Q3ILI6 | Pseudoalteromonas haloplanktis (strain TAC 125) | Putative multidrug resistance protein(AcrB/AcrD/AcrF family) |
| Q3IX11 | Rhodobacter sphaeroides (strain ATCC 17023 / 2.4.1 / NCIB 8253 / DSM158) | Efflux pump membrane transporter |
| Q3J0Q2 | Rhodobacter sphaeroides (strain ATCC 17023 / 2.4.1 / NCIB 8253 / DSM158) | AcrB/AcrD/AcrF family cation/multidrug efflux pump |
| Q3J2H3 | Rhodobacter sphaeroides (strain ATCC 17023 / 2.4.1 / NCIB 8253 / DSM158) | Multidrug/cation efflux pump\| RND superfamily |
| Q3J2M9 | Rhodobacter sphaeroides (strain ATCC 17023 / 2.4.1 / NCIB 8253 / DSM158) | Cation/multidrug efflux pump\| RND superfamily |
| Q3J4A5 | Rhodobacter sphaeroides (strain ATCC 17023 / 2.4.1 / NCIB 8253 / DSM158) | AcrB/AcrD/AcrF multidrug efflux pump |
| Q3J715 | Nitrosococcus oceani (strain ATCC 19707 / BCRC 17464 / NCIMB 11848 /C-107) | Acriflavin resistance protein |
| Q3J9P1 | Nitrosococcus oceani (strain ATCC 19707 / BCRC 17464 / NCIMB 11848 /C-107) | Acriflavin resistance protein |
| Q3JA51 | Nitrosococcus oceani (strain ATCC 19707 / BCRC 17464 / NCIMB 11848 /C-107) | Efflux pump membrane transporter |
| Q3JAX7 | Nitrosococcus oceani (strain ATCC 19707 / BCRC 17464 / NCIMB 11848 /C-107) | Heavy metal efflux pump |
| Q3JBG1 | Nitrosococcus oceani (strain ATCC 19707 / BCRC 17464 / NCIMB 11848 /C-107) | Heavy metal efflux pump |
| Q3JC12 | Nitrosococcus oceani (strain ATCC 19707 / BCRC 17464 / NCIMB 11848 /C-107) | Efflux pump membrane transporter |
| Q3JEC3 | Nitrosococcus oceani (strain ATCC 19707 / BCRC 17464 / NCIMB 11848 /C-107) | Heavy metal efflux pump |
| Q3JEL4 | Nitrosococcus oceani (strain ATCC 19707 / BCRC 17464 / NCIMB 11848 /C-107) | Acriflavin resistance protein |
| Q3JER0 | Nitrosococcus oceani (strain ATCC 19707 / BCRC 17464 / NCIMB 11848 /C-107) | Heavy metal efflux pump |
| Q3JHF5 | Burkholderia pseudomallei (strain 1710b) | Efflux pump membrane transporter |
| Q3JMG5 | Burkholderia pseudomallei (strain 1710b) | Hydrophobe/amphiphile efflux family protein |
| Q3JML3 | Burkholderia pseudomallei (strain 1710b) | Heavy metal efflux pump CzcA |
| Q3JQU5 | Burkholderia pseudomallei (strain 1710b) | AcrB/AcrD/AcrF family protein |
| Q3JRW2 | Burkholderia pseudomallei (strain 1710b) | AcrB/AcrD/AcrF family protein |
| Q3JSK0 | Burkholderia pseudomallei (strain 1710b) | Efflux pump membrane transporter |
| Q3JU49 | Burkholderia pseudomallei (strain 1710b) | AcrB/AcrD/AcrF family protein |
| Q3JVH2 | Burkholderia pseudomallei (strain 1710b) | Efflux pump membrane transporter |
| Q3JWX5 | Burkholderia pseudomallei (strain 1710b) | Heavy metal efflux pump\| CzcA family |
| Q3K755 | Pseudomonas fluorescens (strain Pf0-1) | Putative AcrB/AcrD/AcrF family membrane protein |
| Q3K7M4 | Pseudomonas fluorescens (strain Pf0-1) | Integral membrane component of membrane efflux system |
| Q3KA45 | Pseudomonas fluorescens (strain Pf0-1) | Cobalt-zinc-cadmium resistance membrane protein |
| Q3KC70 | Pseudomonas fluorescens (strain Pf0-1) | Efflux pump membrane transporter |
| Q3KCK7 | Pseudomonas fluorescens (strain Pf0-1) | Efflux pump membrane transporter |
| Q3KCV6 | Pseudomonas fluorescens (strain Pf0-1) | Efflux pump membrane transporter |
| Q3KD91 | Pseudomonas fluorescens (strain Pf0-1) | Multidrug efflux system transmembrane protein |
| Q3KD92 | Pseudomonas fluorescens (strain Pf0-1) | Multidrug efflux system transmembrane protein |
| Q3KDC9 | Pseudomonas fluorescens (strain Pf0-1) | Putative efflux protein |
| Q3KDL7 | Pseudomonas fluorescens (strain Pf0-1) | Heavy metal RND efflux transporter\| CzcA family |
| Q3KGT4 | Pseudomonas fluorescens (strain Pf0-1) | Efflux pump membrane transporter |
| Q3KHD3 | Pseudomonas fluorescens (strain Pf0-1) | Putative transport-related membrane protein |
| Q3KIF5 | Pseudomonas fluorescens (strain Pf0-1) | Integral membrane component of multidrug efflux system |
| Q3KJT0 | Pseudomonas fluorescens (strain Pf0-1) | Putative transport-related membrane protein |
| Q3M3V4 | Anabaena variabilis (strain ATCC 29413 / PCC 7937) | Acriflavin resistance protein |
| Q3M6E3 | Anabaena variabilis (strain ATCC 29413 / PCC 7937) | Hydrophobe/amphiphile efflux-1 HAE1 |
| Q3MA27 | Anabaena variabilis (strain ATCC 29413 / PCC 7937) | Acriflavin resistance protein |
| Q3RC69 | Xylella fastidiosa Dixon. | Efflux pump membrane transporter |
| Q3RC79 | Xylella fastidiosa Dixon. | Acriflavin resistance protein |
| Q3RDM8 | Xylella fastidiosa Dixon. | Acriflavin resistance protein |
| Q3SFX2 | Thiobacillus denitrificans (strain ATCC 25259) | Probable transmembrane drug efflux protein |
| Q3SGA0 | Thiobacillus denitrificans (strain ATCC 25259) | Acriflavin resistance protein |
| Q3SGH8 | Thiobacillus denitrificans (strain ATCC 25259) | Probable RND efflux transporter |
| Q3SI34 | Thiobacillus denitrificans (strain ATCC 25259) | Heavy metal efflux pump CzcA |
| Q3SJ00 | Thiobacillus denitrificans (strain ATCC 25259) | Efflux pump membrane transporter |
| Q3SJ81 | Thiobacillus denitrificans (strain ATCC 25259) | Heavy metal efflux pump CzcA |
| Q3SJ87 | Thiobacillus denitrificans (strain ATCC 25259) | Heavy metal efflux pump CzcA |
| Q3SKD0 | Thiobacillus denitrificans (strain ATCC 25259) | Heavy metal efflux pump CzcA |
| Q3SME9 | Thiobacillus denitrificans (strain ATCC 25259) | Heavy metal efflux pump CzcA |
| Q3SMW3 | Nitrobacter winogradskyi (strain ATCC 25391 / DSM 10237 / CIP 104748 /NCIMB 11846 / Nb-255) | Heavy metal efflux pump CzcA |
| Q3SN60 | Nitrobacter winogradskyi (strain ATCC 25391 / DSM 10237 / CIP 104748 /NCIMB 11846 / Nb-255) | Acriflavin resistance protein |
| Q3SNI0 | Nitrobacter winogradskyi (strain ATCC 25391 / DSM 10237 / CIP 104748 /NCIMB 11846 / Nb-255) | Heavy metal efflux pump CzcA |
| Q3SNZ7 | Nitrobacter winogradskyi (strain ATCC 25391 / DSM 10237 / CIP 104748 /NCIMB 11846 / Nb-255) | Efflux pump membrane transporter |
| Q3SQA4 | Nitrobacter winogradskyi (strain ATCC 25391 / DSM 10237 / CIP 104748 /NCIMB 11846 / Nb-255) | Acriflavin resistance protein |
| Q3SSF8 | Nitrobacter winogradskyi (strain ATCC 25391 / DSM 10237 / CIP 104748 /NCIMB 11846 / Nb-255) | Acriflavin resistance protein |
| Q3SSF9 | Nitrobacter winogradskyi (strain ATCC 25391 / DSM 10237 / CIP 104748 /NCIMB 11846 / Nb-255) | Acriflavin resistance protein |
| Q3SSM3 | Nitrobacter winogradskyi (strain ATCC 25391 / DSM 10237 / CIP 104748 /NCIMB 11846 / Nb-255) | Heavy metal efflux pump CzcA |
| Q3ST65 | Nitrobacter winogradskyi (strain ATCC 25391 / DSM 10237 / CIP 104748 /NCIMB 11846 / Nb-255) | Acriflavin resistance protein |
| Q3YRZ9 | Ehrlichia canis (strain Jake) | Acriflavin resistance protein |
| Q3YWI8 | Shigella sonnei (strain Ss046) | Efflux pump membrane transporter |
| Q3YZ83 | Shigella sonnei (strain Ss046) | Efflux pump membrane transporter |
| Q3Z0C8 | Shigella sonnei (strain Ss046) | Multidrug resistance protein MdtC |
| Q3Z0C9 | Shigella sonnei (strain Ss046) | Multidrug resistance protein MdtB |
| Q3Z4L7 | Shigella sonnei (strain Ss046) | Putative inner membrane component for iron transport |
| Q3Z4T7 | Shigella sonnei (strain Ss046) | Efflux pump membrane transporter |
| Q44586 | Alcaligenes xylosoxydans xylosoxydans | Nickel-cobalt-cadmium resistance protein NccA |
| Q46MN5 | Cupriavidus necator (strain JMP 134 / LMG 1197) | Efflux pump membrane transporter |
| Q46PD4 | Cupriavidus necator (strain JMP 134 / LMG 1197) | Heavy metal efflux pump CzcA |
| Q46PF0 | Cupriavidus necator (strain JMP 134 / LMG 1197) | Acriflavin resistance protein |
| Q46T47 | Cupriavidus necator (strain JMP 134 / LMG 1197) | Efflux pump membrane transporter |
| Q46TT9 | Cupriavidus necator (strain JMP 134 / LMG 1197) | Efflux pump membrane transporter |
| Q46U60 | Cupriavidus necator (strain JMP 134 / LMG 1197) | Heavy metal efflux pump CzcA |
| Q46UM2 | Cupriavidus necator (strain JMP 134 / LMG 1197) | Heavy metal efflux pump CzcA |
| Q46VH3 | Cupriavidus necator (strain JMP 134 / LMG 1197) | Heavy metal efflux pump CzcA |
| Q46VN5 | Cupriavidus necator (strain JMP 134 / LMG 1197) | Efflux pump membrane transporter |
| Q46VQ1 | Cupriavidus necator (strain JMP 134 / LMG 1197) | Acriflavin resistance protein |
| Q46WR1 | Cupriavidus necator (strain JMP 134 / LMG 1197) | Efflux pump membrane transporter |
| Q470K2 | Cupriavidus necator (strain JMP 134 / LMG 1197) | Acriflavin resistance protein |
| Q472E0 | Cupriavidus necator (strain JMP 134 / LMG 1197) | Acriflavin resistance protein |
| Q474Q1 | Cupriavidus necator (strain JMP 134 / LMG 1197) | Acriflavin resistance protein |
| Q475I0 | Cupriavidus necator (strain JMP 134 / LMG 1197) | Acriflavin resistance protein |
| Q479C5 | Dechloromonas aromatica (strain RCB) | Efflux pump membrane transporter |
| Q479J1 | Dechloromonas aromatica (strain RCB) | Acriflavin resistance protein |
| Q47AQ9 | Dechloromonas aromatica (strain RCB) | Acriflavin resistance protein |
| Q47BU3 | Dechloromonas aromatica (strain RCB) | Acriflavin resistance protein |
| Q47CA0 | Dechloromonas aromatica (strain RCB) | Heavy metal efflux pump CzcA |
| Q47CS5 | Dechloromonas aromatica (strain RCB) | Heavy metal efflux pump CzcA |
| Q47CT4 | Dechloromonas aromatica (strain RCB) | Heavy metal efflux pump CzcA |
| Q47D66 | Dechloromonas aromatica (strain RCB) | Heavy metal efflux pump CzcA |
| Q47DS9 | Dechloromonas aromatica (strain RCB) | Heavy metal efflux pump CzcA |
| Q47DU2 | Dechloromonas aromatica (strain RCB) | Heavy metal efflux pump CzcA |
| Q47E30 | Dechloromonas aromatica (strain RCB) | Efflux pump membrane transporter |
| Q47EW0 | Dechloromonas aromatica (strain RCB) | Heavy metal efflux pump CzcA |
| Q47GU7 | Dechloromonas aromatica (strain RCB) | Heavy metal efflux pump CzcA |
| Q47IW5 | Dechloromonas aromatica (strain RCB) | Acriflavin resistance protein |
| Q47JC6 | Dechloromonas aromatica (strain RCB) | Heavy metal efflux pump CzcA |
| Q47MJ1 | Thermobifida fusca (strain YX) | Putative integral membrane efflux protein |
| Q47U52 | Colwellia psychrerythraea (strain 34H / ATCC BAA-681) | AcrB/AcrD/AcrF family protein |
| Q47UM5 | Colwellia psychrerythraea (strain 34H / ATCC BAA-681) | Cation efflux system protein CusA |
| Q47V77 | Colwellia psychrerythraea (strain 34H / ATCC BAA-681) | AcrB/AcrD/AcrF family protein |
| Q47VA5 | Colwellia psychrerythraea (strain 34H / ATCC BAA-681) | AcrB/AcrD/AcrF family protein |
| Q47VP1 | Colwellia psychrerythraea (strain 34H / ATCC BAA-681) | AcrB/AcrD/AcrF family protein |
| Q47X86 | Colwellia psychrerythraea (strain 34H / ATCC BAA-681) | AcrB/AcrD/AcrF family protein |
| Q480E7 | Colwellia psychrerythraea (strain 34H / ATCC BAA-681) | Heavy metal efflux pump\| CzcA family |
| Q480Y8 | Colwellia psychrerythraea (strain 34H / ATCC BAA-681) | AcrB/AcrD/AcrF family protein |
| Q483S5 | Colwellia psychrerythraea (strain 34H / ATCC BAA-681) | AcrB/AcrD/AcrF family protein |
| Q483S7 | Colwellia psychrerythraea (strain 34H / ATCC BAA-681) | Putative RND efflux system protein |
| Q484D7 | Colwellia psychrerythraea (strain 34H / ATCC BAA-681) | AcrB/AcrD/AcrF family protein |
| Q485I6 | Colwellia psychrerythraea (strain 34H / ATCC BAA-681) | Efflux pump membrane transporter |
| Q486B7 | Colwellia psychrerythraea (strain 34H / ATCC BAA-681) | Efflux pump membrane transporter |
| Q48815 | Legionella pneumophila. | Protein HelA |
| Q488L9 | Colwellia psychrerythraea (strain 34H / ATCC BAA-681) | AcrB/AcrD/AcrF family protein |
| Q48A59 | Colwellia psychrerythraea (strain 34H / ATCC BAA-681) | AcrB/AcrD/AcrF family protein |
| Q48CG6 | Pseudomonas savastanoi pv. phaseolicola (strain 1448A / Race 6) | Cation efflux family protein |
| Q48EP3 | Pseudomonas savastanoi pv. phaseolicola (strain 1448A / Race 6) | Efflux pump membrane transporter |
| Q48HB1 | Pseudomonas savastanoi pv. phaseolicola (strain 1448A / Race 6) | Transporter\| AcrB/AcrD/AcrF family |
| Q48HP4 | Pseudomonas savastanoi pv. phaseolicola (strain 1448A / Race 6) | Efflux pump membrane transporter |
| Q48IE8 | Pseudomonas savastanoi pv. phaseolicola (strain 1448A / Race 6) | Multidrug RND efflux transporter\| permease protein MdtC |
| Q48IE9 | Pseudomonas savastanoi pv. phaseolicola (strain 1448A / Race 6) | Multidrug RND efflux transporter\| permease protein MdtB |
| Q48J50 | Pseudomonas savastanoi pv. phaseolicola (strain 1448A / Race 6) | Efflux pump membrane transporter |
| Q48JE7 | Pseudomonas savastanoi pv. phaseolicola (strain 1448A / Race 6) | Efflux pump membrane transporter |
| Q48MA9 | Pseudomonas savastanoi pv. phaseolicola (strain 1448A / Race 6) | RND efflux transporter\| AcrB/AcrD/AcrF family |
| Q48NJ4 | Pseudomonas savastanoi pv. phaseolicola (strain 1448A / Race 6) | RND efflux transporter\| hydrophobe/amphiphile efflux-1 (HAE1) family |
| Q48PN7 | Pseudomonas savastanoi pv. phaseolicola (strain 1448A / Race 6) | RND efflux transporter\| hydrophobe/amphiphile efflux-1 (HAE1) family |
| Q49ZH9 | Staphylococcus saprophyticus subsp. saprophyticus (strain ATCC 15305 /DSM 20229 / NCIMB 8711 / NCTC 7292 / S-41) | Putative cation multidrug efflux pump |
| Q4BYX0 | Crocosphaera watsonii WH 8501. | Hydrophobe/amphiphile efflux-1 HAE1 |
| Q4C067 | Crocosphaera watsonii WH 8501. | Hydrophobe/amphiphile efflux-1 HAE1 |
| Q4ECJ9 | Wolbachia endosymbiont of Drosophila ananassae. | MMPL family protein |
| Q4FP77 | Pelagibacter ubique (strain HTCC1062) | AcrB/AcrD/AcrF family protein (Acriflavin resistance) |
| Q4FPX8 | Psychrobacter arcticus (strain DSM 17307 / 273-4) | RND superfamily multidrug efflux pump |
| Q4FRD4 | Psychrobacter arcticus (strain DSM 17307 / 273-4) | Efflux pump membrane transporter |
| Q4K638 | Pseudomonas fluorescens (strain ATCC BAA-477 / NRRL B-23932 / Pf-5) | Cobalt/zinc/cadmium resistance protein CzcA |
| Q4K6K5 | Pseudomonas fluorescens (strain ATCC BAA-477 / NRRL B-23932 / Pf-5) | RND transporter\| heavy metal efflux (HME) family\| permease protein |
| Q4KAL4 | Pseudomonas fluorescens (strain ATCC BAA-477 / NRRL B-23932 / Pf-5) | RND transporter\| HAE1 family |
| Q4KBK7 | Pseudomonas fluorescens (strain ATCC BAA-477 / NRRL B-23932 / Pf-5) | Efflux pump membrane transporter |
| Q4KBN7 | Pseudomonas fluorescens (strain ATCC BAA-477 / NRRL B-23932 / Pf-5) | Efflux pump membrane transporter |
| Q4KCR6 | Pseudomonas fluorescens (strain ATCC BAA-477 / NRRL B-23932 / Pf-5) | Multidrug RND efflux transporter\| permease protein MdtC |
| Q4KCR7 | Pseudomonas fluorescens (strain ATCC BAA-477 / NRRL B-23932 / Pf-5) | Multidrug RND efflux transporter\| permease protein MdtB |
| Q4KDL8 | Pseudomonas fluorescens (strain ATCC BAA-477 / NRRL B-23932 / Pf-5) | RND transporter\| heavy metal efflux (HME) family\| permease protein |
| Q4KH23 | Pseudomonas fluorescens (strain ATCC BAA-477 / NRRL B-23932 / Pf-5) | Efflux pump membrane transporter |
| Q4KHJ4 | Pseudomonas fluorescens (strain ATCC BAA-477 / NRRL B-23932 / Pf-5) | RND transporter\| hydrophobe/amphiphile efflux-1 (HAE1) family\| permease protein |
| Q4KHX4 | Pseudomonas fluorescens (strain ATCC BAA-477 / NRRL B-23932 / Pf-5) | RND transporter\| HAE1 family |
| Q4KK49 | Pseudomonas fluorescens (strain ATCC BAA-477 / NRRL B-23932 / Pf-5) | RND transporter\| hydrophobe/amphiphile efflux-1 (HAE1) family\| permease protein |
| Q4L8C5 | Staphylococcus haemolyticus (strain JCSC1435) | Uncharacterized protein |
| Q4LDT6 | Pseudomonas aeruginosa. | Efflux pump membrane transporter |
| Q4LDT8 | Pseudomonas aeruginosa. | RND multidrug efflux transporter MexN |
| Q4QM13 | Haemophilus influenzae (strain 86-028NP) | Predicted cation/multidrug efflux pump |
| Q4UKH1 | Rickettsia felis (strain ATCC VR-1525 / URRWXCal2) | Hydrophobe/amphiphile efflux-1 HAE1 family protein |
| Q4VSJ4 | Burkholderia glumae | Probable RND efflux transporter |
| Q4ZLZ2 | Pseudomonas syringae pv. syringae (strain B728a) | Heavy metal efflux pump CzcA |
| Q4ZP84 | Pseudomonas syringae pv. syringae (strain B728a) | Efflux pump membrane transporter |
| Q4ZRQ8 | Pseudomonas syringae pv. syringae (strain B728a) | Acriflavin resistance protein |
| Q4ZS70 | Pseudomonas syringae pv. syringae (strain B728a) | Efflux pump membrane transporter |
| Q4ZSH1 | Pseudomonas syringae pv. syringae (strain B728a) | Efflux pump membrane transporter |
| Q4ZT61 | Pseudomonas syringae pv. syringae (strain B728a) | Acriflavin resistance protein |
| Q4ZTJ9 | Pseudomonas syringae pv. syringae (strain B728a) | Acriflavin resistance protein |
| Q4ZTK0 | Pseudomonas syringae pv. syringae (strain B728a) | Acriflavin resistance protein |
| Q4ZU47 | Pseudomonas syringae pv. syringae (strain B728a) | Efflux pump membrane transporter |
| Q4ZUD5 | Pseudomonas syringae pv. syringae (strain B728a) | Acriflavin resistance protein |
| Q4ZXE0 | Pseudomonas syringae pv. syringae (strain B728a) | Acriflavin resistance protein |
| Q4ZZK4 | Pseudomonas syringae pv. syringae (strain B728a) | Acriflavin resistance protein |
| Q51073 | Neisseria gonorrhoeae. | Efflux pump membrane transporter |
| Q51396 | Pseudomonas aeruginosa. | Efflux pump membrane transporter |
| Q55584 | Synechocystis sp. (strain PCC 6803 / Kazusa) | Cation or drug efflux system protein |
| Q55935 | Synechocystis sp. (strain PCC 6803 / Kazusa) | Cation or drug efflux system protein |
| Q57124 | Haemophilus influenzae (strain ATCC 51907 / DSM 11121 / KW20 / Rd) | Uncharacterized transporter HI_0895 |
| Q579D3 | Brucella abortus biovar 1 (strain 9-941) | AcrB/AcrD/AcrF multidrug efflux protein |
| Q57D49 | Brucella abortus biovar 1 (strain 9-941) | AcrB/AcrD/AcrF multidrug efflux protein |
| Q57F66 | Brucella abortus biovar 1 (strain 9-941) | Efflux pump membrane transporter |
| Q57F78 | Brucella abortus biovar 1 (strain 9-941) | AcrB/AcrD/AcrF multidrug efflux protein |
| Q57J78 | Salmonella choleraesuis (strain SC-B67) | Efflux pump membrane transporter |
| Q57LN0 | Salmonella choleraesuis (strain SC-B67) | Efflux pump membrane transporter |
| Q57MM4 | Salmonella choleraesuis (strain SC-B67) | Multidrug resistance protein MdtC |
| Q57MM5 | Salmonella choleraesuis (strain SC-B67) | Multidrug resistance protein MdtB |
| Q57S88 | Salmonella choleraesuis (strain SC-B67) | Efflux pump membrane transporter |
| Q58AF4 | Cupriavidus metallidurans (strain ATCC 43123 / DSM 2839 / NBRC 102507/ CH34) | SilA\| pump of the three components proton antiporter cation efflux system involved in silver\| copper resistance |
| Q58AG2 | Cupriavidus metallidurans (strain ATCC 43123 / DSM 2839 / NBRC 102507/ CH34) | NccA\| three components proton antiporter cation efflux system\| cation efflux pump |
| Q5DYC7 | Aliivibrio fischeri (strain ATCC 700601 / ES114) | Efflux pump membrane transporter |
| Q5DZ19 | Aliivibrio fischeri (strain ATCC 700601 / ES114) | Efflux pump membrane transporter |
| Q5E0D1 | Aliivibrio fischeri (strain ATCC 700601 / ES114) | Copper/silver efflux system\| membrane component |
| Q5E0L9 | Aliivibrio fischeri (strain ATCC 700601 / ES114) | Acriflavin resistance plasma membrane protein |
| Q5E1S7 | Aliivibrio fischeri (strain ATCC 700601 / ES114) | Acriflavin resistance plasma membrane protein |
| Q5E2W9 | Aliivibrio fischeri (strain ATCC 700601 / ES114) | Acriflavin resistance plasma membrane protein |
| Q5E4H0 | Aliivibrio fischeri (strain ATCC 700601 / ES114) | Acriflavin resistance plasma membrane protein |
| Q5E5L7 | Aliivibrio fischeri (strain ATCC 700601 / ES114) | Acriflavin resistance plasma membrane protein |
| Q5E853 | Aliivibrio fischeri (strain ATCC 700601 / ES114) | Acriflavin resistance plasma membrane protein |
| Q5F725 | Neisseria gonorrhoeae (strain ATCC 700825 / FA 1090) | Efflux pump membrane transporter |
| Q5FGX1 | Ehrlichia ruminantium (strain Gardel) | Probable aminoglycoside efflux pump (Acriflavine resistance protein D) |
| Q5FSA3 | Gluconobacter oxydans (strain 621H) | Cation efflux system protein CzcA |
| Q5FT29 | Gluconobacter oxydans (strain 621H) | Efflux pump membrane transporter |
| Q5FTG4 | Gluconobacter oxydans (strain 621H) | Heavy-metal ion transporter HelA |
| Q5FTX0 | Gluconobacter oxydans (strain 621H) | Putative transport transmembrane protein |
| Q5FU14 | Gluconobacter oxydans (strain 621H) | Acriflavin resistance protein D |
| Q5G7J3 | Rhizobium etli. | Efflux pump membrane transporter |
| Q5GV29 | Xanthomonas oryzae pv. oryzae (strain KACC10331 / KXO85) | Acriflavin resistance protein |
| Q5GXC3 | Xanthomonas oryzae pv. oryzae (strain KACC10331 / KXO85) | Acriflavin resistance protein |
| Q5GZ12 | Xanthomonas oryzae pv. oryzae (strain KACC10331 / KXO85) | Efflux pump membrane transporter |
| Q5H2K4 | Xanthomonas oryzae pv. oryzae (strain KACC10331 / KXO85) | Acriflavin resistance protein |
| Q5HLY7 | Staphylococcus epidermidis (strain ATCC 35984 / RP62A) | AcrB/AcrD/AcrF family protein |
| Q5IS02 | Morganella morganii | Efflux pump membrane transporter |
| Q5KWT2 | Geobacillus kaustophilus (strain HTA426) | Hypothetical conserved protein |
| Q5L7H0 | Bacteroides fragilis (strain ATCC 25285 / DSM 2151 / JCM 11019 /NCTC 9343) | Putative transmembrane AcrB/D/F-family transporter |
| Q5L8F0 | Bacteroides fragilis (strain ATCC 25285 / DSM 2151 / JCM 11019 /NCTC 9343) | Putative transmembrane Acr-type transport protein |
| Q5L8Q3 | Bacteroides fragilis (strain ATCC 25285 / DSM 2151 / JCM 11019 /NCTC 9343) | Putative transport related membrane protein |
| Q5L990 | Bacteroides fragilis (strain ATCC 25285 / DSM 2151 / JCM 11019 /NCTC 9343) | Putative transport-related\| membrane protein |
| Q5L9M7 | Bacteroides fragilis (strain ATCC 25285 / DSM 2151 / JCM 11019 /NCTC 9343) | Putative metal resistance related trasport membrane protein |
| Q5LA33 | Bacteroides fragilis (strain ATCC 25285 / DSM 2151 / JCM 11019 /NCTC 9343) | Putative multidrug resistance/siderophore transport related\| membrane protein |
| Q5LAI6 | Bacteroides fragilis (strain ATCC 25285 / DSM 2151 / JCM 11019 /NCTC 9343) | Putative transport-related membrane protein |
| Q5LB81 | Bacteroides fragilis (strain ATCC 25285 / DSM 2151 / JCM 11019 /NCTC 9343) | Putative aminoglycoside efflux pump |
| Q5LCI8 | Bacteroides fragilis (strain ATCC 25285 / DSM 2151 / JCM 11019 /NCTC 9343) | Putative drug resistance transport-related membrane protein |
| Q5LD76 | Bacteroides fragilis (strain ATCC 25285 / DSM 2151 / JCM 11019 /NCTC 9343) | Putative cation efflux-related membrane protein |
| Q5LDG2 | Bacteroides fragilis (strain ATCC 25285 / DSM 2151 / JCM 11019 /NCTC 9343) | Putative cation transport related membrane protein |
| Q5LHM2 | Bacteroides fragilis (strain ATCC 25285 / DSM 2151 / JCM 11019 /NCTC 9343) | Putative AcrB/AcrD/AcrF family efflux transporter |
| Q5LIU3 | Bacteroides fragilis (strain ATCC 25285 / DSM 2151 / JCM 11019 /NCTC 9343) | Putative AcrB/AcrD family RND transport protein |
| Q5LT89 | Ruegeria pomeroyi (strain ATCC 700808 / DSM 15171 / DSS-3) | Transporter\| AcrB/AcrD/AcrF family |
| Q5LTL7 | Ruegeria pomeroyi (strain ATCC 700808 / DSM 15171 / DSS-3) | Efflux pump membrane transporter |
| Q5LUY1 | Ruegeria pomeroyi (strain ATCC 700808 / DSM 15171 / DSS-3) | Transporter\| AcrB/AcrD/AcrF family |
| Q5LX87 | Ruegeria pomeroyi (strain ATCC 700808 / DSM 15171 / DSS-3) | Transporter\| AcrB/AcrD/AcrF family |
| Q5NIG7 | Francisella tularensis subsp. tularensis (strain SCHU S4 / Schu 4) | Transporter AcrB/AcrD/AcrF family |
| Q5NMA8 | Zymomonas mobilis subsp. mobilis (strain ATCC 31821 / ZM4 / CP4) | Acriflavin resistance protein |
| Q5NQU7 | Zymomonas mobilis subsp. mobilis (strain ATCC 31821 / ZM4 / CP4) | Efflux pump membrane transporter |
| Q5NXR2 | Aromatoleum aromaticum (strain EbN1) | Cation/multidrug efflux pump protein |
| Q5P2W3 | Aromatoleum aromaticum (strain EbN1) | Probable cation efflux system protein CZCA |
| Q5P2Z3 | Aromatoleum aromaticum (strain EbN1) | Predicted acriflavin resistance protein |
| Q5P5T6 | Aromatoleum aromaticum (strain EbN1) | Cation efflux system protein |
| Q5P649 | Aromatoleum aromaticum (strain EbN1) | Efflux pump membrane transporter |
| Q5P6P0 | Aromatoleum aromaticum (strain EbN1) | Probable RND efflux transporter |
| Q5PDW7 | Salmonella paratyphi A (strain ATCC 9150 / SARB42) | Multidrug resistance protein MdtB |
| Q5PDW8 | Salmonella paratyphi A (strain ATCC 9150 / SARB42) | Multidrug resistance protein MdtC |
| Q5QUF0 | Idiomarina loihiensis (strain ATCC BAA-735 / DSM 15497 / L2-TR) | Co/Zn/Cd efflux system membrane component |
| Q5QVV8 | Idiomarina loihiensis (strain ATCC BAA-735 / DSM 15497 / L2-TR) | RND family efflux transporter |
| Q5QWZ8 | Idiomarina loihiensis (strain ATCC BAA-735 / DSM 15497 / L2-TR) | RND family efflux transporter |
| Q5QYY6 | Idiomarina loihiensis (strain ATCC BAA-735 / DSM 15497 / L2-TR) | RND family efflux transporter |
| Q5R021 | Idiomarina loihiensis (strain ATCC BAA-735 / DSM 15497 / L2-TR) | Probable Co/Zn/Cd efflux system membrane component |
| Q5R024 | Idiomarina loihiensis (strain ATCC BAA-735 / DSM 15497 / L2-TR) | RND family efflux transporter |
| Q5R0E7 | Idiomarina loihiensis (strain ATCC BAA-735 / DSM 15497 / L2-TR) | Efflux pump membrane transporter |
| Q5R0R1 | Idiomarina loihiensis (strain ATCC BAA-735 / DSM 15497 / L2-TR) | RND family efflux transporter |
| Q5R0W3 | Idiomarina loihiensis (strain ATCC BAA-735 / DSM 15497 / L2-TR) | Metal efflux system membrane component (Silver efflux pump related) |
| Q5R0W8 | Idiomarina loihiensis (strain ATCC BAA-735 / DSM 15497 / L2-TR) | Metal efflux system membrane component (Silver efflux pump related) |
| Q5R138 | Idiomarina loihiensis (strain ATCC BAA-735 / DSM 15497 / L2-TR) | Co/Zn/Cd efflux system membrane component |
| Q5WCH2 | Bacillus clausii (strain KSM-K16) | AcrB/AcrD/AcrF family cation/multidrug efflux pump |
| Q5WTT9 | Legionella pneumophila (strain Lens) | Efflux pump membrane transporter |
| Q5WUR4 | Legionella pneumophila (strain Lens) | Efflux pump membrane transporter |
| Q5WUV4 | Legionella pneumophila (strain Lens) | Chemiosmotic efflux system protein A-like protein |
| Q5WXP7 | Legionella pneumophila (strain Lens) | HelA protein |
| Q5WYH9 | Legionella pneumophila (strain Lens) | Uncharacterized protein |
| Q5ZSK5 | Legionella pneumophila subsp. pneumophila (strain Philadelphia 1 /ATCC 33152 / DSM 7513) | Efflux pump membrane transporter |
| Q5ZTI4 | Legionella pneumophila subsp. pneumophila (strain Philadelphia 1 /ATCC 33152 / DSM 7513) | Efflux pump membrane transporter |
| Q5ZTM7 | Legionella pneumophila subsp. pneumophila (strain Philadelphia 1 /ATCC 33152 / DSM 7513) | Chemiosmotic efflux system protein A (CzcA) |
| Q5ZWI9 | Legionella pneumophila subsp. pneumophila (strain Philadelphia 1 /ATCC 33152 / DSM 7513) | Chemiosmotic efflux system B protein A |
| Q5ZWQ6 | Legionella pneumophila subsp. pneumophila (strain Philadelphia 1 /ATCC 33152 / DSM 7513) | Chemiosmotic efflux system protein A-like protein |
| Q5ZWR5 | Legionella pneumophila subsp. pneumophila (strain Philadelphia 1 /ATCC 33152 / DSM 7513) | Chemiosmotic efflux system B protein A |
| Q5ZWS7 | Legionella pneumophila subsp. pneumophila (strain Philadelphia 1 /ATCC 33152 / DSM 7513) | Cobalt/zinc/cadmium efflux RND transporter\| permease protein HelA |
| Q5ZXL1 | Legionella pneumophila subsp. pneumophila (strain Philadelphia 1 /ATCC 33152 / DSM 7513) | Multidrug resistance protein |
| Q603J0 | Methylococcus capsulatus (strain ATCC 33009 / NCIMB 11132 / Bath) | AcrB/AcrD/AcrF family protein |
| Q603S9 | Methylococcus capsulatus (strain ATCC 33009 / NCIMB 11132 / Bath) | Heavy metal efflux pump\| CzcA family |
| Q605G0 | Methylococcus capsulatus (strain ATCC 33009 / NCIMB 11132 / Bath) | Heavy metal efflux pump\| CzcA family |
| Q605L6 | Methylococcus capsulatus (strain ATCC 33009 / NCIMB 11132 / Bath) | Heavy metal efflux pump\| CzcA family |
| Q605P8 | Methylococcus capsulatus (strain ATCC 33009 / NCIMB 11132 / Bath) | Efflux pump membrane transporter |
| Q605X2 | Methylococcus capsulatus (strain ATCC 33009 / NCIMB 11132 / Bath) | AcrB/AcrD/AcrF family protein |
| Q605Z1 | Methylococcus capsulatus (strain ATCC 33009 / NCIMB 11132 / Bath) | AcrB/AcrD/AcrF family protein |
| Q606T0 | Methylococcus capsulatus (strain ATCC 33009 / NCIMB 11132 / Bath) | AcrB/AcrD/AcrF family protein |
| Q607J2 | Methylococcus capsulatus (strain ATCC 33009 / NCIMB 11132 / Bath) | Heavy metal efflux pump\| CzcA family |
| Q607N7 | Methylococcus capsulatus (strain ATCC 33009 / NCIMB 11132 / Bath) | AcrB/AcrD/AcrF family protein |
| Q608A0 | Methylococcus capsulatus (strain ATCC 33009 / NCIMB 11132 / Bath) | Efflux pump membrane transporter |
| Q608X6 | Methylococcus capsulatus (strain ATCC 33009 / NCIMB 11132 / Bath) | Efflux pump membrane transporter |
| Q609D7 | Methylococcus capsulatus (strain ATCC 33009 / NCIMB 11132 / Bath) | Heavy metal efflux pump\| CzcA family |
| Q609J5 | Methylococcus capsulatus (strain ATCC 33009 / NCIMB 11132 / Bath) | Heavy metal efflux pump\| CzcA family |
| Q60A90 | Methylococcus capsulatus (strain ATCC 33009 / NCIMB 11132 / Bath) | Heavy metal efflux pump\| CzcA family |
| Q60CD0 | Methylococcus capsulatus (strain ATCC 33009 / NCIMB 11132 / Bath) | AcrB/AcrD/AcrF family protein |
| Q60CM7 | Methylococcus capsulatus (strain ATCC 33009 / NCIMB 11132 / Bath) | AcrB/AcrD/AcrF family protein |
| Q630G7 | Bacillus cereus (strain ZK / E33L) | Conserved transporter possible acriflavin resistance protein |
| Q63FS7 | Bacillus cereus (strain ZK / E33L) | Acriflavin resistance protein |
| Q63L89 | Burkholderia pseudomallei (strain K96243) | Putative RND efflux transporter |
| Q63LH4 | Burkholderia pseudomallei (strain K96243) | Putative cation efflux system protein |
| Q63NK6 | Burkholderia pseudomallei (strain K96243) | Efflux pump membrane transporter |
| Q63ST6 | Burkholderia pseudomallei (strain K96243) | Putative AcrB/AcrD/AcrF family membrane protein |
| Q63U14 | Burkholderia pseudomallei (strain K96243) | Efflux pump membrane transporter |
| Q63UM9 | Burkholderia pseudomallei (strain K96243) | Putative drug-resistance cell envelope-related protein |
| Q63VH8 | Burkholderia pseudomallei (strain K96243) | Putative transport system\| membrane protein |
| Q63WS7 | Burkholderia pseudomallei (strain K96243) | Efflux pump membrane transporter |
| Q63Y79 | Burkholderia pseudomallei (strain K96243) | Putative cation efflux system protein |
| Q64MM9 | Bacteroides fragilis (strain YCH46) | AcrB/D/F family transporter |
| Q64NQ0 | Bacteroides fragilis (strain YCH46) | Putative cation efflux transporter |
| Q64PF1 | Bacteroides fragilis (strain YCH46) | AcrB/AcrD/AcrF family cation efflux system protein |
| Q64PW6 | Bacteroides fragilis (strain YCH46) | AcrB/AcrD/AcrF family cation efflux system protein |
| Q64QE5 | Bacteroides fragilis (strain YCH46) | AcrB/AcrD family multidrug resistance protein |
| Q64RM7 | Bacteroides fragilis (strain YCH46) | Putative aminoglycoside efflux pump |
| Q64TN7 | Bacteroides fragilis (strain YCH46) | Putative cation efflux pump |
| Q64U98 | Bacteroides fragilis (strain YCH46) | Cation efflux system protein CzcA |
| Q64UJ0 | Bacteroides fragilis (strain YCH46) | CzcA family cation efflux system protein |
| Q64YJ6 | Bacteroides fragilis (strain YCH46) | Multidrug efflux membrane fusion protein |
| Q64ZY7 | Bacteroides fragilis (strain YCH46) | AcrB/AcrD family multidrug resistance protein |
| Q65MP2 | Bacillus licheniformis (strain ATCC 14580 / DSM 13 / JCM 2505 / NBRC12200 / NCIMB 9375 / NRRL NRS-1264 / Gibson 46) | Swarming and motility protein SwrC |
| Q65QR6 | Mannheimia succiniciproducens (strain MBEL55E) | AcrB protein |
| Q65VE8 | Mannheimia succiniciproducens (strain MBEL55E) | AcrB protein |
| Q662M2 | Borreliella bavariensis (strain ATCC BAA-2496 / DSM 23469 / PBi) | Acriflavine resistance protein |
| Q666F2 | Yersinia pseudotuberculosis serotype I (strain IP32953) | Efflux pump membrane transporter |
| Q668C5 | Yersinia pseudotuberculosis serotype I (strain IP32953) | Multidrug resistance protein MdtC |
| Q668C6 | Yersinia pseudotuberculosis serotype I (strain IP32953) | Multidrug resistance protein MdtB |
| Q668H5 | Yersinia pseudotuberculosis serotype I (strain IP32953) | Efflux pump membrane transporter |
| Q66DR0 | Yersinia pseudotuberculosis serotype I (strain IP32953) | Efflux pump membrane transporter |
| Q66EX5 | Yersinia pseudotuberculosis serotype I (strain IP32953) | Putative heavy metal/multi-drug efflux protein\| RND family |
| Q66F48 | Yersinia pseudotuberculosis serotype I (strain IP32953) | Efflux pump membrane transporter |
| Q67J93 | Symbiobacterium thermophilum (strain T / IAM 14863) | AcrB family membrane transport protein |
| Q68XJ7 | Rickettsia typhi (strain ATCC VR-144 / Wilmington) | Acriflavin resistance protein D |
| Q69HW2 | Escherichia coli. | Efflux pump membrane transporter |
| Q6AJB4 | Desulfotalea psychrophila (strain LSv54 / DSM 12343) | Related to cobalt-zinc-cadmium resistance protein (CzcA) |
| Q6ALC4 | Desulfotalea psychrophila (strain LSv54 / DSM 12343) | Related to multidrug-efflux transport protein |
| Q6AMJ9 | Desulfotalea psychrophila (strain LSv54 / DSM 12343) | Efflux pump membrane transporter |
| Q6ARC4 | Desulfotalea psychrophila (strain LSv54 / DSM 12343) | Probable cation efflux system protein (CzcA) |
| Q6CZM0 | Pectobacterium atrosepticum (strain SCRI 1043 / ATCC BAA-672) | Efflux pump membrane transporter |
| Q6D1J9 | Pectobacterium atrosepticum (strain SCRI 1043 / ATCC BAA-672) | Efflux pump membrane transporter |
| Q6D2B0 | Pectobacterium atrosepticum (strain SCRI 1043 / ATCC BAA-672) | Multidrug resistance protein MdtC |
| Q6D2B1 | Pectobacterium atrosepticum (strain SCRI 1043 / ATCC BAA-672) | Multidrug resistance protein MdtB |
| Q6D315 | Pectobacterium atrosepticum (strain SCRI 1043 / ATCC BAA-672) | Putative efflux protein |
| Q6D7E2 | Pectobacterium atrosepticum (strain SCRI 1043 / ATCC BAA-672) | Cation efflux system protein |
| Q6D806 | Pectobacterium atrosepticum (strain SCRI 1043 / ATCC BAA-672) | Efflux pump membrane transporter |
| Q6EMD9 | Escherichia coli. | Cu(+)/Ag(+) efflux RND transporter permease subunit SilA |
| Q6F6Q9 | Acinetobacter baylyi (strain ATCC 33305 / BD413 / ADP1) | Putative efflux transporter causing drug resistance (Acr family) |
| Q6F786 | Acinetobacter baylyi (strain ATCC 33305 / BD413 / ADP1) | Multidrug transport protein (RND family) |
| Q6F787 | Acinetobacter baylyi (strain ATCC 33305 / BD413 / ADP1) | Multidrug transport protein\| outer membrane (RND family) |
| Q6F7C5 | Acinetobacter baylyi (strain ATCC 33305 / BD413 / ADP1) | RND divalent metal cation efflux transporter |
| Q6F8F6 | Acinetobacter baylyi (strain ATCC 33305 / BD413 / ADP1) | Efflux pump membrane transporter |
| Q6F8P8 | Acinetobacter baylyi (strain ATCC 33305 / BD413 / ADP1) | Efflux pump membrane transporter |
| Q6FD21 | Acinetobacter baylyi (strain ATCC 33305 / BD413 / ADP1) | Putative multidrug transporter |
| Q6FD22 | Acinetobacter baylyi (strain ATCC 33305 / BD413 / ADP1) | Putative RND efflux transporter |
| Q6FE22 | Acinetobacter baylyi (strain ATCC 33305 / BD413 / ADP1) | Nodulation protein |
| Q6HAK2 | Bacillus thuringiensis subsp. konkukian (strain 97-27) | Conserved transporter possible acriflavin resistance protein |
| Q6HN96 | Bacillus thuringiensis subsp. konkukian (strain 97-27) | Acriflavin resistance protein |
| Q6IVS4 | uncultured gamma proteobacterium eBACHOT4E07. | Predicted cation efflux system |
| Q6KAY1 | Stenotrophomonas maltophilia | Efflux pump membrane transporter |
| Q6KAZ0 | Stenotrophomonas maltophilia | Efflux pump membrane transporter |
| Q6KAZ6 | Stenotrophomonas maltophilia | Efflux pump membrane transporter |
| Q6LGP3 | Photobacterium profundum (strain SS9) | Putative transporter\| AcrB/D/F family |
| Q6LGT2 | Photobacterium profundum (strain SS9) | Efflux pump membrane transporter |
| Q6LIY4 | Photobacterium profundum (strain SS9) | Efflux pump membrane transporter |
| Q6LNM7 | Photobacterium profundum (strain SS9) | Putative multidrug resistance protein |
| Q6LPI6 | Photobacterium profundum (strain SS9) | Putative AcrB\| Cation/multidrug efflux pump |
| Q6LPS3 | Photobacterium profundum (strain SS9) | Hypothetical transporter\| AcrB/D/F family |
| Q6LTG2 | Photobacterium profundum (strain SS9) | Putative cation efflux system transmembrane protein |
| Q6LV21 | Photobacterium profundum (strain SS9) | Putative Cation/multidrug efflux pump |
| Q6LVZ3 | Photobacterium profundum (strain SS9) | Putative multidrug resistance protein |
| Q6MDJ2 | Protochlamydia amoebophila (strain UWE25) | Uncharacterized protein |
| Q6MDS4 | Protochlamydia amoebophila (strain UWE25) | Uncharacterized protein |
| Q6MEI7 | Protochlamydia amoebophila (strain UWE25) | Uncharacterized protein |
| Q6MIU0 | Bdellovibrio bacteriovorus (strain ATCC 15356 / DSM 50701 / NCIB 9529/ HD100) | Efflux transporter |
| Q6ML00 | Bdellovibrio bacteriovorus (strain ATCC 15356 / DSM 50701 / NCIB 9529/ HD100) | Cation efflux system protein\| AcrB/AcrD/AcrF family protein |
| Q6MM46 | Bdellovibrio bacteriovorus (strain ATCC 15356 / DSM 50701 / NCIB 9529/ HD100) | NolG efflux transporter |
| Q6MM88 | Bdellovibrio bacteriovorus (strain ATCC 15356 / DSM 50701 / NCIB 9529/ HD100) | NolG efflux transporter |
| Q6MNU1 | Bdellovibrio bacteriovorus (strain ATCC 15356 / DSM 50701 / NCIB 9529/ HD100) | Acriflavin resistance protein |
| Q6MP63 | Bdellovibrio bacteriovorus (strain ATCC 15356 / DSM 50701 / NCIB 9529/ HD100) | Acriflavin resistance protein |
| Q6MPG6 | Bdellovibrio bacteriovorus (strain ATCC 15356 / DSM 50701 / NCIB 9529/ HD100) | Acriflavin resistance protein |
| Q6MXQ0 | Serratia marcescens. | Putative cation efflux system protein (Silver resistance) |
| Q6N0T1 | Rhodopseudomonas palustris (strain ATCC BAA-98 / CGA009) | Putative efflux transporter |
| Q6N1C7 | Rhodopseudomonas palustris (strain ATCC BAA-98 / CGA009) | Putative heavy metal cation efflux system protein |
| Q6N1J1 | Rhodopseudomonas palustris (strain ATCC BAA-98 / CGA009) | Putative RND efflux transporter |
| Q6N2F5 | Rhodopseudomonas palustris (strain ATCC BAA-98 / CGA009) | Efflux pump membrane transporter |
| Q6N2Z4 | Rhodopseudomonas palustris (strain ATCC BAA-98 / CGA009) | Efflux pump membrane transporter |
| Q6N3B9 | Rhodopseudomonas palustris (strain ATCC BAA-98 / CGA009) | Putative RND efflux transporter |
| Q6N457 | Rhodopseudomonas palustris (strain ATCC BAA-98 / CGA009) | RND efflux transporter |
| Q6N5L8 | Rhodopseudomonas palustris (strain ATCC BAA-98 / CGA009) | Efflux pump membrane transporter |
| Q6N682 | Rhodopseudomonas palustris (strain ATCC BAA-98 / CGA009) | Efflux pump membrane transporter |
| Q6N6N9 | Rhodopseudomonas palustris (strain ATCC BAA-98 / CGA009) | Possible RND efflux transporter |
| Q6N6P0 | Rhodopseudomonas palustris (strain ATCC BAA-98 / CGA009) | Possible RND efflux transporter |
| Q6N787 | Rhodopseudomonas palustris (strain ATCC BAA-98 / CGA009) | Efflux pump membrane transporter |
| Q6N848 | Rhodopseudomonas palustris (strain ATCC BAA-98 / CGA009) | RND divalent metal cation efflux transporter CzcA |
| Q6N8D5 | Rhodopseudomonas palustris (strain ATCC BAA-98 / CGA009) | RND efflux transporter |
| Q6N8E2 | Rhodopseudomonas palustris (strain ATCC BAA-98 / CGA009) | RND efflux transporter |
| Q6N8U5 | Rhodopseudomonas palustris (strain ATCC BAA-98 / CGA009) | Putative RND divalent metal cation efflux transporter CzcA |
| Q6N8Z7 | Rhodopseudomonas palustris (strain ATCC BAA-98 / CGA009) | Putative cation efflux system protein |
| Q6N9P2 | Rhodopseudomonas palustris (strain ATCC BAA-98 / CGA009) | Efflux pump membrane transporter |
| Q6N9W6 | Rhodopseudomonas palustris (strain ATCC BAA-98 / CGA009) | Putative inner membrane component for iron transport |
| Q6NB09 | Rhodopseudomonas palustris (strain ATCC BAA-98 / CGA009) | Putative transporter\| AcrB/D/F family Cation efflux system protein |
| Q6NDK9 | Rhodopseudomonas palustris (strain ATCC BAA-98 / CGA009) | Efflux pump membrane transporter |
| Q6PKZ7 | Campylobacter coli. | Efflux pump membrane transporter |
| Q6Q918 | uncultured marine gamma proteobacterium EBAC20E09. | Predicted cation efflux system |
| Q6SGZ1 | uncultured marine bacterium 443. | Efflux transporter\| RND family\| outer membrane subunit\| putative |
| Q6SH26 | uncultured marine bacterium 442. | Multidrug efflux transporter\| AcrB/AcrD/AcrF family |
| Q6U5N9 | Klebsiella pneumoniae CG43. | SilA |
| Q6V6X8 | Pseudomonas fluorescens. | Efflux pump membrane transporter |
| Q6VV68 | Burkholderia pseudomallei | Efflux pump membrane transporter |
| Q6W1F3 | Sinorhizobium fredii (strain NBRC 101917 / NGR234) | Acriflavin resistance plasma membrane protein |
| Q6YRW1 | Synechocystis sp. (strain PCC 6803 / Kazusa) | Slr6043 protein |
| Q71UZ6 | Pseudomonas stutzeri | Efflux pump membrane transporter |
| Q725M1 | Desulfovibrio vulgaris (strain Hildenborough / ATCC 29579 / DSM 644 /NCIMB 8303) | Efflux pump membrane transporter |
| Q727N9 | Desulfovibrio vulgaris (strain Hildenborough / ATCC 29579 / DSM 644 /NCIMB 8303) | Efflux pump membrane transporter |
| Q72EY0 | Desulfovibrio vulgaris (strain Hildenborough / ATCC 29579 / DSM 644 /NCIMB 8303) | AcrB/AcrD/AcrF family protein |
| Q72G02 | Desulfovibrio vulgaris (strain Hildenborough / ATCC 29579 / DSM 644 /NCIMB 8303) | Efflux pump membrane transporter |
| Q72MQ9 | Leptospira interrogans serogroup Icterohaemorrhagiae serovarcopenhageni (strain Fiocruz L1-130) | Acriflavin resistance |
| Q72MU9 | Leptospira interrogans serogroup Icterohaemorrhagiae serovarcopenhageni (strain Fiocruz L1-130) | Acriflavin resistance |
| Q72MW5 | Leptospira interrogans serogroup Icterohaemorrhagiae serovarcopenhageni (strain Fiocruz L1-130) | Acriflavin resistance |
| Q72Q88 | Leptospira interrogans serogroup Icterohaemorrhagiae serovarcopenhageni (strain Fiocruz L1-130) | Heavy metal efflux pump |
| Q72V12 | Leptospira interrogans serogroup Icterohaemorrhagiae serovarcopenhageni (strain Fiocruz L1-130) | Acriflavin resistance |
| Q72X10 | Bacillus cereus (strain ATCC 10987 / NRS 248) | Transporter\| AcrB/AcrD/AcrF family |
| Q73DC5 | Bacillus cereus (strain ATCC 10987 / NRS 248) | Transporter\| AcrB/AcrD/AcrF family |
| Q73FK8 | Wolbachia pipientis wMel. | Multidrug resistance protein D |
| Q746W8 | Geobacter sulfurreducens (strain ATCC 51573 / DSM 12127 / PCA) | Heavy metal efflux pump\| RND family\| inner membrane protein\| CzcA family |
| Q749G1 | Geobacter sulfurreducens (strain ATCC 51573 / DSM 12127 / PCA) | Efflux pump\| RND family\| inner membrane protein\| AcrB/AcrD/AcrF family |
| Q749P6 | Geobacter sulfurreducens (strain ATCC 51573 / DSM 12127 / PCA) | Efflux pump membrane transporter |
| Q749S8 | Geobacter sulfurreducens (strain ATCC 51573 / DSM 12127 / PCA) | Efflux pump\| RND family\| inner and outer membrane proteins |
| Q74BA6 | Geobacter sulfurreducens (strain ATCC 51573 / DSM 12127 / PCA) | Efflux pump\| RND family\| inner membrane protein |
| Q74CR1 | Geobacter sulfurreducens (strain ATCC 51573 / DSM 12127 / PCA) | Efflux pump\| RND family\| inner membrane protein\| AcrB/AcrD/AcrF family |
| Q74DI4 | Geobacter sulfurreducens (strain ATCC 51573 / DSM 12127 / PCA) | Metal ion efflux pump\| RND family\| inner membrane protein |
| Q74EX9 | Geobacter sulfurreducens (strain ATCC 51573 / DSM 12127 / PCA) | Efflux pump\| RND family\| inner membrane protein |
| Q74G55 | Geobacter sulfurreducens (strain ATCC 51573 / DSM 12127 / PCA) | Efflux pump\| RND family\| inner membrane protein |
| Q79MP3 | Serratia marcescens subsp. marcescens. | Multidrug resistance protein MdtC |
| Q7ACM1 | Escherichia coli O157:H7. | Multidrug resistance protein MdtC |
| Q7B054 | Cupriavidus metallidurans. | CnrA protein |
| Q7M912 | Wolinella succinogenes (strain ATCC 29543 / DSM 1740 / LMG 7466 /NCTC 11488 / FDC 602W) | RND PUMP PROTEIN |
| Q7M9I0 | Wolinella succinogenes (strain ATCC 29543 / DSM 1740 / LMG 7466 /NCTC 11488 / FDC 602W) | Efflux pump membrane transporter |
| Q7M9P9 | Wolinella succinogenes (strain ATCC 29543 / DSM 1740 / LMG 7466 /NCTC 11488 / FDC 602W) | Efflux pump membrane transporter |
| Q7MCH1 | Vibrio vulnificus (strain YJ016) | Putative multidrug resistance protein |
| Q7MCR8 | Vibrio vulnificus (strain YJ016) | Putative silver efflux pump |
| Q7MDF8 | Vibrio vulnificus (strain YJ016) | Efflux pump membrane transporter |
| Q7MDY8 | Vibrio vulnificus (strain YJ016) | Transporter\| AcrB/D/F family |
| Q7MEG9 | Vibrio vulnificus (strain YJ016) | Transporter\| AcrB/D/F family |
| Q7MEY5 | Vibrio vulnificus (strain YJ016) | Transporter\| AcrB/D/F family |
| Q7MG13 | Vibrio vulnificus (strain YJ016) | Uncharacterized protein |
| Q7MHZ2 | Vibrio vulnificus (strain YJ016) | Putative multidrug resistance protein |
| Q7MLN0 | Vibrio vulnificus (strain YJ016) | Transporter\| AcrB/D/F family |
| Q7MME3 | Vibrio vulnificus (strain YJ016) | Putative multidrug resistance protein |
| Q7MQH1 | Vibrio vulnificus (strain YJ016) | Putative multidrug resistance protein |
| Q7MWQ6 | Porphyromonas gingivalis (strain ATCC BAA-308 / W83) | AcrB/AcrD/AcrF family protein |
| Q7MXU3 | Porphyromonas gingivalis (strain ATCC BAA-308 / W83) | Heavy metal efflux pump\| CzcA family |
| Q7N0N0 | Photorhabdus luminescens subsp. laumondii (strain DSM 15139 / CIP105565 / TT01) | Efflux pump membrane transporter |
| Q7N3E1 | Photorhabdus luminescens subsp. laumondii (strain DSM 15139 / CIP105565 / TT01) | Multidrug resistance protein MdtC |
| Q7N3E2 | Photorhabdus luminescens subsp. laumondii (strain DSM 15139 / CIP105565 / TT01) | Multidrug resistance protein MdtB |
| Q7N8G7 | Photorhabdus luminescens subsp. laumondii (strain DSM 15139 / CIP105565 / TT01) | Uncharacterized protein |
| Q7NCY7 | Gloeobacter violaceus (strain ATCC 29082 / PCC 7421) | RND multidrug efflux transporter |
| Q7NDR2 | Gloeobacter violaceus (strain ATCC 29082 / PCC 7421) | Gll4170 protein |
| Q7NE52 | Gloeobacter violaceus (strain ATCC 29082 / PCC 7421) | Glr4028 protein |
| Q7NE92 | Gloeobacter violaceus (strain ATCC 29082 / PCC 7421) | RND multidrug efflux transporter |
| Q7NFA7 | Gloeobacter violaceus (strain ATCC 29082 / PCC 7421) | Gll3619 protein |
| Q7NHP1 | Gloeobacter violaceus (strain ATCC 29082 / PCC 7421) | AcrB/AcrD/AcrF family protein |
| Q7NJ01 | Gloeobacter violaceus (strain ATCC 29082 / PCC 7421) | Cation efflux system protein |
| Q7NL29 | Gloeobacter violaceus (strain ATCC 29082 / PCC 7421) | AcrB/AcrD/AcrF family protein |
| Q7NM91 | Gloeobacter violaceus (strain ATCC 29082 / PCC 7421) | Gll0876 protein |
| Q7NMG0 | Gloeobacter violaceus (strain ATCC 29082 / PCC 7421) | AcrB/AcrD/AcrF family protein |
| Q7NNM9 | Gloeobacter violaceus (strain ATCC 29082 / PCC 7421) | Glr0382 protein |
| Q7NNZ4 | Gloeobacter violaceus (strain ATCC 29082 / PCC 7421) | AcrB/AcrD/AcrF family protein |
| Q7NP24 | Gloeobacter violaceus (strain ATCC 29082 / PCC 7421) | AcrB/AcrD/AcrF family protein |
| Q7NR60 | Chromobacterium violaceum (strain ATCC 12472 / DSM 30191 / JCM 1249 /NBRC 12614 / NCIMB 9131 / NCTC 9757) | Probable transmembrane drug efflux protein |
| Q7NRE6 | Chromobacterium violaceum (strain ATCC 12472 / DSM 30191 / JCM 1249 /NBRC 12614 / NCIMB 9131 / NCTC 9757) | Probable multidrug efflux membrane protein |
| Q7NUG4 | Chromobacterium violaceum (strain ATCC 12472 / DSM 30191 / JCM 1249 /NBRC 12614 / NCIMB 9131 / NCTC 9757) | Probable drug efflux pump transmembrane protein |
| Q7NUG5 | Chromobacterium violaceum (strain ATCC 12472 / DSM 30191 / JCM 1249 /NBRC 12614 / NCIMB 9131 / NCTC 9757) | Probable drug efflux pump transmembrane protein |
| Q7NVV1 | Chromobacterium violaceum (strain ATCC 12472 / DSM 30191 / JCM 1249 /NBRC 12614 / NCIMB 9131 / NCTC 9757) | Efflux pump membrane transporter |
| Q7NWL0 | Chromobacterium violaceum (strain ATCC 12472 / DSM 30191 / JCM 1249 /NBRC 12614 / NCIMB 9131 / NCTC 9757) | NolG efflux transporter |
| Q7NXK0 | Chromobacterium violaceum (strain ATCC 12472 / DSM 30191 / JCM 1249 /NBRC 12614 / NCIMB 9131 / NCTC 9757) | Probable multidrug efflux protein |
| Q7P0Y1 | Chromobacterium violaceum (strain ATCC 12472 / DSM 30191 / JCM 1249 /NBRC 12614 / NCIMB 9131 / NCTC 9757) | Efflux pump membrane transporter |
| Q7UDI0 | Shigella flexneri. | Efflux pump membrane transporter |
| Q7UEM8 | Rhodopirellula baltica (strain DSM 10527 / NCIMB 13988 / SH1) | Cation efflux system protein CZCA |
| Q7UH35 | Rhodopirellula baltica (strain DSM 10527 / NCIMB 13988 / SH1) | Predicted cation efflux system (AcrB/AcrD/AcrF family) |
| Q7UJP2 | Rhodopirellula baltica (strain DSM 10527 / NCIMB 13988 / SH1) | Probable multidrug resistance protein |
| Q7UJT6 | Rhodopirellula baltica (strain DSM 10527 / NCIMB 13988 / SH1) | Acriflavine resistance protein B |
| Q7ULF2 | Rhodopirellula baltica (strain DSM 10527 / NCIMB 13988 / SH1) | RND multidrug efflux transporter MexF |
| Q7USF5 | Rhodopirellula baltica (strain DSM 10527 / NCIMB 13988 / SH1) | Cation efflux system protein czcA-1 |
| Q7UZ48 | Rhodopirellula baltica (strain DSM 10527 / NCIMB 13988 / SH1) | Cation efflux system\| AcrB/AcrD/AcrF family |
| Q7VII0 | Helicobacter hepaticus (strain ATCC 51449 / 3B1) | Uncharacterized protein |
| Q7VJM1 | Helicobacter hepaticus (strain ATCC 51449 / 3B1) | SSD domain-containing protein |
| Q7VJR9 | Helicobacter hepaticus (strain ATCC 51449 / 3B1) | Efflux pump membrane transporter |
| Q7VLE5 | Haemophilus ducreyi (strain 35000HP / ATCC 700724) | Acriflavine resistance protein |
| Q7VSV8 | Bordetella pertussis (strain Tohama I / ATCC BAA-589 / NCTC 13251) | AcrB/AcrD/AcrF family protein |
| Q7VSV9 | Bordetella pertussis (strain Tohama I / ATCC BAA-589 / NCTC 13251) | AcrB/AcrD/AcrF family protein |
| Q7VWW1 | Bordetella pertussis (strain Tohama I / ATCC BAA-589 / NCTC 13251) | Efflux pump membrane transporter |
| Q7VZD3 | Bordetella pertussis (strain Tohama I / ATCC BAA-589 / NCTC 13251) | Efflux pump membrane transporter |
| Q7VZR4 | Bordetella pertussis (strain Tohama I / ATCC BAA-589 / NCTC 13251) | Integral membrane component of multidrug efflux system |
| Q7W3S1 | Bordetella parapertussis (strain 12822 / ATCC BAA-587 / NCTC 13253) | AcrB/AcrD/AcrF family protein |
| Q7W3S2 | Bordetella parapertussis (strain 12822 / ATCC BAA-587 / NCTC 13253) | AcrB/AcrD/AcrF family protein |
| Q7W438 | Bordetella parapertussis (strain 12822 / ATCC BAA-587 / NCTC 13253) | Probable membrane protein |
| Q7WAC5 | Bordetella parapertussis (strain 12822 / ATCC BAA-587 / NCTC 13253) | Efflux pump membrane transporter |
| Q7WC93 | Bordetella parapertussis (strain 12822 / ATCC BAA-587 / NCTC 13253) | Efflux pump membrane transporter |
| Q7WSD5 | Serratia marcescens. | Efflux pump membrane transporter |
| Q7WTQ9 | Erwinia amylovora | Efflux pump membrane transporter |
| Q7X364 | uncultured Acidobacteria bacterium. | Putative multidrug resistance pump |
| Q814J5 | Bacillus cereus (strain ATCC 14579 / DSM 31 / JCM 2152 / NBRC 15305 /NCIMB 9373 / NRRL B-3711) | Acriflavin resistance plasma membrane protein |
| Q81HR8 | Bacillus cereus (strain ATCC 14579 / DSM 31 / JCM 2152 / NBRC 15305 /NCIMB 9373 / NRRL B-3711) | Acriflavin resistance plasma membrane protein |
| Q81JL8 | Bacillus anthracis. | Transporter\| AcrB/AcrD/AcrF family |
| Q820K8 | Nitrosomonas europaea (strain ATCC 19718 / CIP 103999 / KCTC 2705 /NBRC 14298) | Acriflavin resistance protein:Heavy metal efflux pump CzcA |
| Q820R2 | Nitrosomonas europaea (strain ATCC 19718 / CIP 103999 / KCTC 2705 /NBRC 14298) | Acriflavin resistance protein:Heavy metal efflux pump CzcA |
| Q820R6 | Nitrosomonas europaea (strain ATCC 19718 / CIP 103999 / KCTC 2705 /NBRC 14298) | Acriflavin resistance protein:Heavy metal efflux pump CzcA |
| Q82AL7 | Streptomyces avermitilis (strain ATCC 31267 / DSM 46492 / JCM 5070 /NBRC 14893 / NCIMB 12804 / NRRL 8165 / MA-4680) | Putative cation/multidrug efflux protein |
| Q82T82 | Nitrosomonas europaea (strain ATCC 19718 / CIP 103999 / KCTC 2705 /NBRC 14298) | Acriflavin resistance protein |
| Q82VH6 | Nitrosomonas europaea (strain ATCC 19718 / CIP 103999 / KCTC 2705 /NBRC 14298) | Efflux pump membrane transporter |
| Q82WK5 | Nitrosomonas europaea (strain ATCC 19718 / CIP 103999 / KCTC 2705 /NBRC 14298) | Acriflavin resistance protein |
| Q82XT4 | Nitrosomonas europaea (strain ATCC 19718 / CIP 103999 / KCTC 2705 /NBRC 14298) | Efflux pump membrane transporter |
| Q82XU2 | Nitrosomonas europaea (strain ATCC 19718 / CIP 103999 / KCTC 2705 /NBRC 14298) | Efflux pump membrane transporter |
| Q83CM1 | Coxiella burnetii (strain RSA 493 / Nine Mile phase I) | Acriflavin resistance plasma membrane protein |
| Q83DD4 | Coxiella burnetii (strain RSA 493 / Nine Mile phase I) | Acriflavin resistance plasma membrane protein |
| Q83DH8 | Coxiella burnetii (strain RSA 493 / Nine Mile phase I) | Acriflavin resistance plasma membrane protein |
| Q83KI4 | Shigella flexneri. | Multidrug resistance protein MdtC |
| Q83SC3 | Shigella flexneri. | Putative inner membrane component for iron transport |
| Q840D3 | Acinetobacter baumannii. | Efflux pump membrane transporter |
| Q849R0 | Pseudomonas putida (strain ATCC 700007 / DSM 6899 / BCRC 17059 / F1) | Probable efflux pump membrane transporter SepB |
| Q84GI9 | Serratia marcescens subsp. marcescens. | Efflux pump membrane transporter |
| Q87BP4 | Xylella fastidiosa (strain Temecula1 / ATCC 700964) | Acriflavin resistance protein |
| Q87DA3 | Xylella fastidiosa (strain Temecula1 / ATCC 700964) | Efflux pump membrane transporter |
| Q87EU7 | Xylella fastidiosa (strain Temecula1 / ATCC 700964) | Acriflavin resistance protein |
| Q87GX5 | Vibrio parahaemolyticus serotype O3:K6 (strain RIMD 2210633) | Efflux pump membrane transporter |
| Q87HZ7 | Vibrio parahaemolyticus serotype O3:K6 (strain RIMD 2210633) | Transporter\| AcrB/D/F family |
| Q87IX6 | Vibrio parahaemolyticus serotype O3:K6 (strain RIMD 2210633) | Putative cation efflux system transmembrane protein |
| Q87IY5 | Vibrio parahaemolyticus serotype O3:K6 (strain RIMD 2210633) | Efflux pump membrane transporter |
| Q87J90 | Vibrio parahaemolyticus serotype O3:K6 (strain RIMD 2210633) | Putative efflux protein |
| Q87JA9 | Vibrio parahaemolyticus serotype O3:K6 (strain RIMD 2210633) | Putative multidrug resistance protein |
| Q87LY6 | Vibrio parahaemolyticus serotype O3:K6 (strain RIMD 2210633) | Putative multidrug resistance protein |
| Q87QH1 | Vibrio parahaemolyticus serotype O3:K6 (strain RIMD 2210633) | Transporter\| AcrB/D/F family |
| Q87QQ7 | Vibrio parahaemolyticus serotype O3:K6 (strain RIMD 2210633) | Efflux pump membrane transporter |
| Q87R57 | Vibrio parahaemolyticus serotype O3:K6 (strain RIMD 2210633) | Putative multidrug resistance protein |
| Q87TN1 | Vibrio parahaemolyticus serotype O3:K6 (strain RIMD 2210633) | Putative multidrug resistance protein |
| Q87UV1 | Pseudomonas syringae pv. tomato (strain ATCC BAA-871 / DC3000) | AcrB/AcrD/AcrF family protein |
| Q87X84 | Pseudomonas syringae pv. tomato (strain ATCC BAA-871 / DC3000) | Efflux pump membrane transporter |
| Q87ZX0 | Pseudomonas syringae pv. tomato (strain ATCC BAA-871 / DC3000) | AcrB/AcrD/AcrF family protein |
| Q880Q4 | Pseudomonas syringae pv. tomato (strain ATCC BAA-871 / DC3000) | Efflux pump membrane transporter |
| Q881X7 | Pseudomonas syringae pv. tomato (strain ATCC BAA-871 / DC3000) | AcrB/AcrD/AcrF family protein |
| Q882N4 | Pseudomonas syringae pv. tomato (strain ATCC BAA-871 / DC3000) | Efflux pump membrane transporter |
| Q887I4 | Pseudomonas syringae pv. tomato (strain ATCC BAA-871 / DC3000) | AcrB/AcrD/AcrF family protein |
| Q889D0 | Pseudomonas syringae pv. tomato (strain ATCC BAA-871 / DC3000) | AcrB/AcrD/AcrF family protein |
| Q88AL5 | Pseudomonas syringae pv. tomato (strain ATCC BAA-871 / DC3000) | Cation efflux family protein |
| Q88BZ6 | Pseudomonas putida (strain ATCC 47054 / DSM 6125 / NCIMB 11950 /KT2440) | Probable copper efflux transporter\| CzcA family |
| Q88CK7 | Pseudomonas putida (strain ATCC 47054 / DSM 6125 / NCIMB 11950 /KT2440) | RND efflux transporter |
| Q88GY2 | Pseudomonas putida (strain ATCC 47054 / DSM 6125 / NCIMB 11950 /KT2440) | Multidrug efflux transport system-membrane subunit |
| Q88HA4 | Pseudomonas putida (strain ATCC 47054 / DSM 6125 / NCIMB 11950 /KT2440) | Efflux pump membrane transporter |
| Q88HD4 | Pseudomonas putida (strain ATCC 47054 / DSM 6125 / NCIMB 11950 /KT2440) | Efflux pump membrane transporter |
| Q88HQ1 | Pseudomonas putida (strain ATCC 47054 / DSM 6125 / NCIMB 11950 /KT2440) | RND efflux transporter |
| Q88J31 | Pseudomonas putida (strain ATCC 47054 / DSM 6125 / NCIMB 11950 /KT2440) | Efflux pump membrane transporter |
| Q88K81 | Pseudomonas putida (strain ATCC 47054 / DSM 6125 / NCIMB 11950 /KT2440) | Cation efflux system protein |
| Q88L70 | Pseudomonas putida (strain ATCC 47054 / DSM 6125 / NCIMB 11950 /KT2440) | Multidrug efflux RND transporter |
| Q88MQ3 | Pseudomonas putida (strain ATCC 47054 / DSM 6125 / NCIMB 11950 /KT2440) | RND efflux transporter |
| Q88N31 | Pseudomonas putida (strain ATCC 47054 / DSM 6125 / NCIMB 11950 /KT2440) | Probable efflux pump membrane transporter TtgB |
| Q88PE4 | Pseudomonas putida (strain ATCC 47054 / DSM 6125 / NCIMB 11950 /KT2440) | Putative Multidrug efflux RND transporter |
| Q88RT6 | Pseudomonas putida (strain ATCC 47054 / DSM 6125 / NCIMB 11950 /KT2440) | Cation efflux system protein |
| Q89DV7 | Bradyrhizobium diazoefficiens (strain JCM 10833 / IAM 13628 / NBRC14792 / USDA 110) | Efflux pump membrane transporter |
| Q89DX5 | Bradyrhizobium diazoefficiens (strain JCM 10833 / IAM 13628 / NBRC14792 / USDA 110) | AcrB/AcrD/AcrF family protein |
| Q89EQ3 | Bradyrhizobium diazoefficiens (strain JCM 10833 / IAM 13628 / NBRC14792 / USDA 110) | AcrB/AcrD/AcrF family protein |
| Q89FH4 | Bradyrhizobium diazoefficiens (strain JCM 10833 / IAM 13628 / NBRC14792 / USDA 110) | Efflux pump membrane transporter |
| Q89I68 | Bradyrhizobium diazoefficiens (strain JCM 10833 / IAM 13628 / NBRC14792 / USDA 110) | AcrB/AcrD/AcrF family cation efflux protein |
| Q89K38 | Bradyrhizobium diazoefficiens (strain JCM 10833 / IAM 13628 / NBRC14792 / USDA 110) | Efflux pump membrane transporter |
| Q89KG8 | Bradyrhizobium diazoefficiens (strain JCM 10833 / IAM 13628 / NBRC14792 / USDA 110) | Blr4937 protein |
| Q89KH2 | Bradyrhizobium diazoefficiens (strain JCM 10833 / IAM 13628 / NBRC14792 / USDA 110) | Blr4933 protein |
| Q89LT5 | Bradyrhizobium diazoefficiens (strain JCM 10833 / IAM 13628 / NBRC14792 / USDA 110) | Blr4458 protein |
| Q89LT6 | Bradyrhizobium diazoefficiens (strain JCM 10833 / IAM 13628 / NBRC14792 / USDA 110) | AcrB/AcrD/AcrF family protein |
| Q89M74 | Bradyrhizobium diazoefficiens (strain JCM 10833 / IAM 13628 / NBRC14792 / USDA 110) | Bll4319 protein |
| Q89MT0 | Bradyrhizobium diazoefficiens (strain JCM 10833 / IAM 13628 / NBRC14792 / USDA 110) | Probale cation efflux system protein |
| Q89NE0 | Bradyrhizobium diazoefficiens (strain JCM 10833 / IAM 13628 / NBRC14792 / USDA 110) | AcrB/AcrD/AcrF family protein |
| Q89NG9 | Bradyrhizobium diazoefficiens (strain JCM 10833 / IAM 13628 / NBRC14792 / USDA 110) | Efflux pump membrane transporter |
| Q89QU2 | Bradyrhizobium diazoefficiens (strain JCM 10833 / IAM 13628 / NBRC14792 / USDA 110) | Cation efflux system protein |
| Q89R38 | Bradyrhizobium diazoefficiens (strain JCM 10833 / IAM 13628 / NBRC14792 / USDA 110) | RagC protein |
| Q89RB1 | Bradyrhizobium diazoefficiens (strain JCM 10833 / IAM 13628 / NBRC14792 / USDA 110) | Blr2861 protein |
| Q89SH7 | Bradyrhizobium diazoefficiens (strain JCM 10833 / IAM 13628 / NBRC14792 / USDA 110) | Cation efflux system protein |
| Q89TZ3 | Bradyrhizobium diazoefficiens (strain JCM 10833 / IAM 13628 / NBRC14792 / USDA 110) | Multidrug resistance protein |
| Q89UA1 | Bradyrhizobium diazoefficiens (strain JCM 10833 / IAM 13628 / NBRC14792 / USDA 110) | Efflux pump membrane transporter |
| Q89VP8 | Bradyrhizobium diazoefficiens (strain JCM 10833 / IAM 13628 / NBRC14792 / USDA 110) | Efflux pump membrane transporter |
| Q89XF8 | Bradyrhizobium diazoefficiens (strain JCM 10833 / IAM 13628 / NBRC14792 / USDA 110) | Acr family transport protein |
| Q89XK8 | Bradyrhizobium diazoefficiens (strain JCM 10833 / IAM 13628 / NBRC14792 / USDA 110) | Cation efflux protein |
| Q89XN1 | Bradyrhizobium diazoefficiens (strain JCM 10833 / IAM 13628 / NBRC14792 / USDA 110) | AcrB/AcrD/AcrF family protein |
| Q89YN7 | Bacteroides thetaiotaomicron (strain ATCC 29148 / DSM 2079 /NCTC 10582 / E50 / VPI-5482) | Cation efflux system protein |
| Q8A0Q2 | Bacteroides thetaiotaomicron (strain ATCC 29148 / DSM 2079 /NCTC 10582 / E50 / VPI-5482) | Cation efflux system protein\| AcrB/AcrD/AcrF family protein |
| Q8A2G7 | Bacteroides thetaiotaomicron (strain ATCC 29148 / DSM 2079 /NCTC 10582 / E50 / VPI-5482) | AcrB/AcrD family multidrug resistance protein |
| Q8A3L3 | Bacteroides thetaiotaomicron (strain ATCC 29148 / DSM 2079 /NCTC 10582 / E50 / VPI-5482) | Multidrug resistance protein mexB (Multidrug-efflux protein) |
| Q8A4B7 | Bacteroides thetaiotaomicron (strain ATCC 29148 / DSM 2079 /NCTC 10582 / E50 / VPI-5482) | Putative cation efflux transporter |
| Q8A5I7 | Bacteroides thetaiotaomicron (strain ATCC 29148 / DSM 2079 /NCTC 10582 / E50 / VPI-5482) | Multidrug efflux membrane fusion protein |
| Q8A647 | Bacteroides thetaiotaomicron (strain ATCC 29148 / DSM 2079 /NCTC 10582 / E50 / VPI-5482) | Cation efflux system protein\| AcrB/AcrD/AcrF family protein |
| Q8A6B8 | Bacteroides thetaiotaomicron (strain ATCC 29148 / DSM 2079 /NCTC 10582 / E50 / VPI-5482) | Multidrug resistance protein\| AcrB/AcrD family |
| Q8A899 | Bacteroides thetaiotaomicron (strain ATCC 29148 / DSM 2079 /NCTC 10582 / E50 / VPI-5482) | Transporter\| AcrB/D/F family |
| Q8A9C9 | Bacteroides thetaiotaomicron (strain ATCC 29148 / DSM 2079 /NCTC 10582 / E50 / VPI-5482) | Putative cation efflux pump |
| Q8A9Y5 | Bacteroides thetaiotaomicron (strain ATCC 29148 / DSM 2079 /NCTC 10582 / E50 / VPI-5482) | Cation efflux system protein czcA |
| Q8AB07 | Bacteroides thetaiotaomicron (strain ATCC 29148 / DSM 2079 /NCTC 10582 / E50 / VPI-5482) | Putative aminoglycoside efflux pump (Acriflavine resistance protein) |
| Q8AB13 | Bacteroides thetaiotaomicron (strain ATCC 29148 / DSM 2079 /NCTC 10582 / E50 / VPI-5482) | Cation efflux system (AcrB/AcrD/AcrF family) |
| Q8CK05 | Streptomyces coelicolor (strain ATCC BAA-471 / A3(2) | Putative integral membrane efflux protein |
| Q8CX78 | Oceanobacillus iheyensis (strain DSM 14371 / CIP 107618 / JCM 11309 /KCTC 3954 / HTE831) | Acriflavine resistance protein (Cation efflux system) |
| Q8DIH0 | Thermosynechococcus elongatus (strain BP-1) | Multidrug efflux transporter |
| Q8DJR3 | Thermosynechococcus elongatus (strain BP-1) | AcrB/AcrD/AcrF family protein |
| Q8E808 | Shewanella oneidensis (strain MR-1) | Copper/silver efflux pump permease component CusA |
| Q8E8H2 | Shewanella oneidensis (strain MR-1) | Efflux pump membrane transporter |
| Q8E8R3 | Shewanella oneidensis (strain MR-1) | Copper/silver efflux pump permease component CusA |
| Q8EA94 | Shewanella oneidensis (strain MR-1) | HAE1 family effllux pump permease component |
| Q8EBL9 | Shewanella oneidensis (strain MR-1) | Efflux pump membrane transporter |
| Q8EBM6 | Shewanella oneidensis (strain MR-1) | HAE1 family efflux pump permease component |
| Q8EC65 | Shewanella oneidensis (strain MR-1) | RND superfamily efflux pump permease component |
| Q8ECN3 | Shewanella oneidensis (strain MR-1) | Thiophosphate efflux pump permease component |
| Q8EFP6 | Shewanella oneidensis (strain MR-1) | RND superfamily efflux pump permease component 2 |
| Q8EFP7 | Shewanella oneidensis (strain MR-1) | RND superfamily efflux pump permease component 1 |
| Q8EFT4 | Shewanella oneidensis (strain MR-1) | HAE1 family efflux pump permease component |
| Q8EI98 | Shewanella oneidensis (strain MR-1) | RND superfamily efflux pump permease component |
| Q8EJE7 | Shewanella oneidensis (strain MR-1) | Heavy metal efflux pump permease component CzcA family |
| Q8EZC8 | Leptospira interrogans serogroup Icterohaemorrhagiae serovar Lai (strain 56601) | Cation/multidrug efflux pump |
| Q8EZI2 | Leptospira interrogans serogroup Icterohaemorrhagiae serovar Lai (strain 56601) | Acriflavine resistance protein |
| Q8EZK7 | Leptospira interrogans serogroup Icterohaemorrhagiae serovar Lai (strain 56601) | Cation/multidrug efflux pump |
| Q8EZW3 | Leptospira interrogans serogroup Icterohaemorrhagiae serovar Lai (strain 56601) | Acriflavine resistance protein |
| Q8F5X3 | Leptospira interrogans serogroup Icterohaemorrhagiae serovar Lai (strain 56601) | Heavy metal efflux pump |
| Q8FCI8 | Escherichia coli O6:H1 (strain CFT073 / ATCC 700928 / UPEC) | Multidrug resistance protein MdtF |
| Q8FG03 | Escherichia coli O6:H1 (strain CFT073 / ATCC 700928 / UPEC) | Multidrug resistance protein MdtC |
| Q8FG04 | Escherichia coli O6:H1 (strain CFT073 / ATCC 700928 / UPEC) | Multidrug resistance protein MdtB |
| Q8FK36 | Escherichia coli O6:H1 (strain CFT073 / ATCC 700928 / UPEC) | Cation efflux system protein CusA |
| Q8FWV9 | Brucella suis biovar 1 (strain 1330) | Efflux pump membrane transporter BepG |
| Q8G2M6 | Brucella suis biovar 1 (strain 1330) | Efflux pump membrane transporter BepE |
| Q8GC83 | Klebsiella aerogenes | Efflux pump membrane transporter |
| Q8GKU1 | Acinetobacter sp. 4365. | Efflux pump membrane transporter |
| Q8KAV4 | Chlorobaculum tepidum (strain ATCC 49652 / DSM 12025 / NBRC 103806 /TLS) | AcrB/AcrD/AcrF family protein |
| Q8KCX0 | Chlorobaculum tepidum (strain ATCC 49652 / DSM 12025 / NBRC 103806 /TLS) | Multidrug resistance protein\| AcrB/AcrD family |
| Q8P3N5 | Xanthomonas campestris pv. campestris (strain ATCC 33913 / DSM 3586 /NCPPB 528 / LMG 568 / P 25) | Cation efflux system protein |
| Q8P4C1 | Xanthomonas campestris pv. campestris (strain ATCC 33913 / DSM 3586 /NCPPB 528 / LMG 568 / P 25) | Acriflavin resistance protein |
| Q8P613 | Xanthomonas campestris pv. campestris (strain ATCC 33913 / DSM 3586 /NCPPB 528 / LMG 568 / P 25) | Acriflavin resistance protein |
| Q8P7C9 | Xanthomonas campestris pv. campestris (strain ATCC 33913 / DSM 3586 /NCPPB 528 / LMG 568 / P 25) | Efflux pump membrane transporter |
| Q8P875 | Xanthomonas campestris pv. campestris (strain ATCC 33913 / DSM 3586 /NCPPB 528 / LMG 568 / P 25) | Efflux pump membrane transporter |
| Q8P8U2 | Xanthomonas campestris pv. campestris (strain ATCC 33913 / DSM 3586 /NCPPB 528 / LMG 568 / P 25) | Transport protein |
| Q8P8U3 | Xanthomonas campestris pv. campestris (strain ATCC 33913 / DSM 3586 /NCPPB 528 / LMG 568 / P 25) | Transport protein |
| Q8PAN9 | Xanthomonas campestris pv. campestris (strain ATCC 33913 / DSM 3586 /NCPPB 528 / LMG 568 / P 25) | Efflux pump membrane transporter |
| Q8PDB8 | Xanthomonas campestris pv. campestris (strain ATCC 33913 / DSM 3586 /NCPPB 528 / LMG 568 / P 25) | Cation efflux system protein |
| Q8PF28 | Xanthomonas axonopodis pv. citri (strain 306) | Cation efflux system protein |
| Q8PFX2 | Xanthomonas axonopodis pv. citri (strain 306) | Acriflavin resistance protein |
| Q8PHD3 | Xanthomonas axonopodis pv. citri (strain 306) | Acriflavin resistance protein |
| Q8PIQ2 | Xanthomonas axonopodis pv. citri (strain 306) | Efflux pump membrane transporter |
| Q8PIU6 | Xanthomonas axonopodis pv. citri (strain 306) | Acriflavin resistance protein |
| Q8PJN1 | Xanthomonas axonopodis pv. citri (strain 306) | Efflux pump membrane transporter |
| Q8PKM4 | Xanthomonas axonopodis pv. citri (strain 306) | Cation efflux system protein |
| Q8PKU6 | Xanthomonas axonopodis pv. citri (strain 306) | Transport protein |
| Q8PKU7 | Xanthomonas axonopodis pv. citri (strain 306) | Transport protein |
| Q8PME6 | Xanthomonas axonopodis pv. citri (strain 306) | Efflux pump membrane transporter |
| Q8PQ89 | Xanthomonas axonopodis pv. citri (strain 306) | Cation efflux system protein |
| Q8PQJ5 | Xanthomonas axonopodis pv. citri (strain 306) | Efflux pump membrane transporter |
| Q8RE51 | Fusobacterium nucleatum subsp. nucleatum (strain ATCC 25586 / CIP101130 / JCM 8532 / LMG 13131) | Acriflavin resistance protein B |
| Q8RG07 | Fusobacterium nucleatum subsp. nucleatum (strain ATCC 25586 / CIP101130 / JCM 8532 / LMG 13131) | Acriflavin resistance protein D |
| Q8RG44 | Fusobacterium nucleatum subsp. nucleatum (strain ATCC 25586 / CIP101130 / JCM 8532 / LMG 13131) | Acriflavin resistance protein B |
| Q8RNP2 | Legionella pneumophila. | Chemiosmotic efflux system B protein A |
| Q8RNQ8 | Legionella pneumophila. | AcrB/AcrD/AcrF family protein |
| Q8RSM1 | uncultured bacterium. | MexD protein |
| Q8RTE4 | Campylobacter jejuni. | Efflux pump membrane transporter |
| Q8VPA8 | Proteus mirabilis. | Efflux pump membrane transporter |
| Q8X3J5 | Escherichia coli O157:H7. | Multidrug resistance protein MdtF |
| Q8X7E2 | Escherichia coli O157:H7. | Efflux pump membrane transporter |
| Q8X7J4 | Escherichia coli O157:H7. | Multidrug resistance protein MdtB |
| Q8XBY1 | Escherichia coli O157:H7. | Cation efflux system protein CusA |
| Q8XD55 | Escherichia coli O157:H7. | Efflux pump membrane transporter |
| Q8XEH2 | Escherichia coli O157:H7. | Efflux pump membrane transporter |
| Q8XPP1 | Ralstonia solanacearum (strain GMI1000) | Probable transport transmembrane protein |
| Q8XQ28 | Ralstonia solanacearum (strain GMI1000) | Probable transporter transmembrane protein |
| Q8XQM3 | Ralstonia solanacearum (strain GMI1000) | Probable drug efflux transmembrane protein |
| Q8XQM4 | Ralstonia solanacearum (strain GMI1000) | Probable drug efflux pump transmembrane protein |
| Q8XQV5 | Ralstonia solanacearum (strain GMI1000) | Efflux pump membrane transporter |
| Q8XR28 | Ralstonia solanacearum (strain GMI1000) | Putative cation efflux system transmembrane protein |
| Q8XRD0 | Ralstonia solanacearum (strain GMI1000) | Probable cation efflux system transmembrane protein |
| Q8XRL3 | Ralstonia solanacearum (strain GMI1000) | Efflux pump membrane transporter |
| Q8XSE6 | Ralstonia solanacearum (strain GMI1000) | Putative cation efflux system transmembrane protein |
| Q8XSI1 | Ralstonia solanacearum (strain GMI1000) | Probable cation efflux system transmembrane protein |
| Q8XT05 | Ralstonia solanacearum (strain GMI1000) | Efflux pump membrane transporter |
| Q8XUI3 | Ralstonia solanacearum (strain GMI1000) | Probable transmembrane drug efflux protein |
| Q8XYV2 | Ralstonia solanacearum (strain GMI1000) | Probable transmembrane drug efflux protein |
| Q8Y3H0 | Ralstonia solanacearum (strain GMI1000) | Efflux pump membrane transporter |
| Q8YCQ5 | Brucella melitensis biotype 1 (strain 16M / ATCC 23456 / NCTC 10094) | Acriflavin resistance protein f |
| Q8YCZ5 | Brucella melitensis biotype 1 (strain 16M / ATCC 23456 / NCTC 10094) | Acriflavin resistance protein d |
| Q8YF77 | Brucella melitensis biotype 1 (strain 16M / ATCC 23456 / NCTC 10094) | Acriflavin resistance protein b |
| Q8YF93 | Brucella melitensis biotype 1 (strain 16M / ATCC 23456 / NCTC 10094) | Efflux pump membrane transporter |
| Q8YHA9 | Brucella melitensis biotype 1 (strain 16M / ATCC 23456 / NCTC 10094) | Acriflavin resistance protein b |
| Q8YLK4 | Nostoc sp. (strain PCC 7120 / SAG 25.82 / UTEX 2576) | Alr5294 protein |
| Q8YSE5 | Nostoc sp. (strain PCC 7120 / SAG 25.82 / UTEX 2576) | RND multidrug efflux transporter |
| Q8YWF7 | Nostoc sp. (strain PCC 7120 / SAG 25.82 / UTEX 2576) | Alr1656 protein |
| Q8Z4S4 | Salmonella typhi. | Efflux pump membrane transporter |
| Q8Z5F6 | Salmonella typhi. | Multidrug resistance protein MdtC |
| Q8Z5F7 | Salmonella typhi. | Multidrug resistance protein MdtB |
| Q8Z8T8 | Salmonella typhi. | Efflux pump membrane transporter |
| Q8ZCV9 | Yersinia pestis. | Multidrug resistance protein MdtC |
| Q8ZCW0 | Yersinia pestis. | Multidrug resistance protein MdtB |
| Q8ZLN4 | Salmonella typhimurium (strain LT2 / SGSC1412 / ATCC 700720) | Efflux pump membrane transporter |
| Q8ZN77 | Salmonella typhimurium (strain LT2 / SGSC1412 / ATCC 700720) | Efflux pump membrane transporter |
| Q8ZNQ1 | Salmonella typhimurium (strain LT2 / SGSC1412 / ATCC 700720) | Multidrug resistance protein MdtC |
| Q8ZNQ2 | Salmonella typhimurium (strain LT2 / SGSC1412 / ATCC 700720) | Multidrug resistance protein MdtB |
| Q8ZRA7 | Salmonella typhimurium (strain LT2 / SGSC1412 / ATCC 700720) | Efflux pump membrane transporter |
| Q8ZRG9 | Salmonella typhimurium (strain LT2 / SGSC1412 / ATCC 700720) | Efflux pump membrane transporter |
| Q8ZS81 | Nostoc sp. (strain PCC 7120 / SAG 25.82 / UTEX 2576) | Cation efflux system protein |
| Q8ZS94 | Nostoc sp. (strain PCC 7120 / SAG 25.82 / UTEX 2576) | Cation efflux system protein |
| Q92J58 | Rickettsia conorii (strain ATCC VR-613 / Malish 7) | Acriflavin resistance protein D |
| Q92M87 | Rhizobium meliloti (strain 1021) | Efflux pump membrane transporter |
| Q92NP7 | Rhizobium meliloti (strain 1021) | Probable acriflavine resistance protein |
| Q92SH0 | Rhizobium meliloti (strain 1021) | Efflux pump membrane transporter |
| Q92T03 | Rhizobium meliloti (strain 1021) | Efflux pump membrane transporter |
| Q92U15 | Rhizobium meliloti (strain 1021) | Efflux pump membrane transporter |
| Q92WK8 | Rhizobium meliloti (strain 1021) | Probable acriflavine family protein |
| Q92Y52 | Rhizobium meliloti (strain 1021) | Cation/multidrug efflux protein |
| Q92YH0 | Rhizobium meliloti (strain 1021) | Efflux pump membrane transporter |
| Q93E19 | Acinetobacter baumannii. | Efflux pump membrane transporter |
| Q93K40 | Klebsiella pneumoniae. | Efflux pump membrane transporter |
| Q93PU4 | Pseudomonas putida (strain DOT-T1E) | Toluene efflux pump membrane transporter TtgH |
| Q93SR9 | Pseudomonas putida | Membrane-bound cation-proton-antiporter CzrA |
| Q986H1 | Mesorhizobium japonicum (strain LMG 29417 / CECT 9101 / MAFF 303099) | Efflux pump membrane transporter |
| Q986L9 | Mesorhizobium japonicum (strain LMG 29417 / CECT 9101 / MAFF 303099) | Component of multidrug efflux system |
| Q988I4 | Mesorhizobium japonicum (strain LMG 29417 / CECT 9101 / MAFF 303099) | Efflux pump membrane transporter |
| Q98B06 | Mesorhizobium japonicum (strain LMG 29417 / CECT 9101 / MAFF 303099) | RND efflux transporter |
| Q98BL7 | Mesorhizobium japonicum (strain LMG 29417 / CECT 9101 / MAFF 303099) | RND efflux transporter |
| Q98FD0 | Mesorhizobium japonicum (strain LMG 29417 / CECT 9101 / MAFF 303099) | Multidrug resistance protein |
| Q98FR6 | Mesorhizobium japonicum (strain LMG 29417 / CECT 9101 / MAFF 303099) | RND efflux transporter |
| Q98GK4 | Mesorhizobium japonicum (strain LMG 29417 / CECT 9101 / MAFF 303099) | Efflux pump membrane transporter |
| Q98IH3 | Mesorhizobium japonicum (strain LMG 29417 / CECT 9101 / MAFF 303099) | Probable RND efflux transporter |
| Q98KL0 | Mesorhizobium japonicum (strain LMG 29417 / CECT 9101 / MAFF 303099) | Probable RND efflux transporter |
| Q9A3K6 | Caulobacter vibrioides (strain ATCC 19089 / CB15) | AcrB/AcrD/AcrF family protein |
| Q9A4V1 | Caulobacter vibrioides (strain ATCC 19089 / CB15) | Metal ion efflux RND protein family |
| Q9A5Q7 | Caulobacter vibrioides (strain ATCC 19089 / CB15) | AcrB/AcrD/AcrF family protein |
| Q9A7D5 | Caulobacter vibrioides (strain ATCC 19089 / CB15) | AcrB/AcrD/AcrF family protein |
| Q9A8Z1 | Caulobacter vibrioides (strain ATCC 19089 / CB15) | AcrB/AcrD/AcrF family protein |
| Q9AA04 | Caulobacter vibrioides (strain ATCC 19089 / CB15) | Efflux pump membrane transporter |
| Q9AEG1 | Klebsiella aerogenes | Efflux pump membrane transporter |
| Q9AG05 | Wolbachia sp. subsp. Drosophila simulans (strain wRi) | Multidrug resistance protein D |
| Q9ALR2 | Pseudomonas fluorescens. | CztA |
| Q9CLS7 | Pasteurella multocida (strain Pm70) | AcrB |
| Q9F240 | Stenotrophomonas maltophilia | Efflux pump membrane transporter |
| Q9F7M0 | Gamma-proteobacterium EBAC31A08. | Predicted cation efflux system (AcrB/AcrD/AcrF family) |
| Q9F8V7 | Rhizobium radiobacter | Efflux pump membrane transporter |
| Q9HVI9 | Pseudomonas aeruginosa (strain ATCC 15692 / DSM 22644 / CIP 104116 /JCM 14847 / LMG 12228 / 1C / PRS 101 / PAO1) | Efflux pump membrane transporter |
| Q9HW27 | Pseudomonas aeruginosa (strain ATCC 15692 / DSM 22644 / CIP 104116 /JCM 14847 / LMG 12228 / 1C / PRS 101 / PAO1) | Probable Resistance-Nodulation-Cell Division (RND) efflux transporter |
| Q9HWH4 | Pseudomonas aeruginosa (strain ATCC 15692 / DSM 22644 / CIP 104116 /JCM 14847 / LMG 12228 / 1C / PRS 101 / PAO1) | Probable Resistance-Nodulation-Cell Division (RND) efflux transporter |
| Q9HXW4 | Pseudomonas aeruginosa (strain ATCC 15692 / DSM 22644 / CIP 104116 /JCM 14847 / LMG 12228 / 1C / PRS 101 / PAO1) | Probable Resistance-Nodulation-Cell Division (RND) efflux transporter |
| Q9HY87 | Pseudomonas aeruginosa (strain ATCC 15692 / DSM 22644 / CIP 104116 /JCM 14847 / LMG 12228 / 1C / PRS 101 / PAO1) | Efflux pump membrane transporter |
| Q9I0V6 | Pseudomonas aeruginosa (strain ATCC 15692 / DSM 22644 / CIP 104116 /JCM 14847 / LMG 12228 / 1C / PRS 101 / PAO1) | Probable Resistance-Nodulation-Cell Division (RND) efflux transporter |
| Q9I0V7 | Pseudomonas aeruginosa (strain ATCC 15692 / DSM 22644 / CIP 104116 /JCM 14847 / LMG 12228 / 1C / PRS 101 / PAO1) | Probable Resistance-Nodulation-Cell Division (RND) efflux transporter |
| Q9I0W2 | Pseudomonas aeruginosa (strain ATCC 15692 / DSM 22644 / CIP 104116 /JCM 14847 / LMG 12228 / 1C / PRS 101 / PAO1) | Resistance-Nodulation-Cell Division (RND) divalent metal cation efflux transporter CzcA |
| Q9I0Y8 | Pseudomonas aeruginosa (strain ATCC 15692 / DSM 22644 / CIP 104116 /JCM 14847 / LMG 12228 / 1C / PRS 101 / PAO1) | Efflux pump membrane transporter |
| Q9I3R1 | Pseudomonas aeruginosa (strain ATCC 15692 / DSM 22644 / CIP 104116 /JCM 14847 / LMG 12228 / 1C / PRS 101 / PAO1) | Probable Resistance-Nodulation-Cell Division (RND) efflux transporter |
| Q9I6X4 | Pseudomonas aeruginosa (strain ATCC 15692 / DSM 22644 / CIP 104116 /JCM 14847 / LMG 12228 / 1C / PRS 101 / PAO1) | Probable Resistance-Nodulation-Cell Division (RND) efflux transporter |
| Q9JY67 | Neisseria meningitidis serogroup B (strain MC58) | Efflux pump membrane transporter |
| Q9K6B3 | Bacillus halodurans (strain ATCC BAA-125 / DSM 18197 / FERM 7344 / JCM9153 / C-125) | Cation efflux system |
| Q9KJC2 | Pseudomonas putida | Antibiotic efflux pump membrane transporter ArpB |
| Q9KLV3 | Vibrio cholerae serotype O1 (strain ATCC 39315 / El Tor Inaba N16961) | Transporter\| AcrB/D/F family |
| Q9KR85 | Vibrio cholerae serotype O1 (strain ATCC 39315 / El Tor Inaba N16961) | Transporter\| AcrB/D/F family |
| Q9KRG9 | Vibrio cholerae serotype O1 (strain ATCC 39315 / El Tor Inaba N16961) | Transporter\| AcrB/D/F family |
| Q9KTI8 | Vibrio cholerae serotype O1 (strain ATCC 39315 / El Tor Inaba N16961) | Multidrug resistance protein\| putative |
| Q9KU94 | Vibrio cholerae serotype O1 (strain ATCC 39315 / El Tor Inaba N16961) | Multidrug resistance protein\| putative |
| Q9KVI2 | Vibrio cholerae serotype O1 (strain ATCC 39315 / El Tor Inaba N16961) | Multidrug resistance protein\| putative |
| Q9KW65 | Pseudomonas syringae. | ORFF protein |
| Q9KWV4 | Pseudomonas putida (strain DOT-T1E) | Toluene efflux pump membrane transporter TtgE |
| Q9PAV9 | Xylella fastidiosa (strain 9a5c) | Acriflavin resistance protein |
| Q9PBP6 | Xylella fastidiosa (strain 9a5c) | Efflux pump membrane transporter |
| Q9PBQ7 | Xylella fastidiosa (strain 9a5c) | Cation efflux system protein |
| Q9PGQ5 | Xylella fastidiosa (strain 9a5c) | Acriflavin resistance protein |
| Q9RBY8 | Stenotrophomonas maltophilia | Efflux pump membrane transporter |
| Q9RG59 | Pseudomonas aeruginosa. | Efflux pump membrane transporter |
| Q9RLI8 | Pseudomonas aeruginosa. | CzrA protein |
| Q9RQG6 | Staphylococcus aureus. | AcrB/AcrD/AcrF family protein |
| Q9WYK5 | Thermotoga maritima (strain ATCC 43589 / MSB8 / DSM 3109 / JCM 10099) | Cation efflux system protein\| putative |
| Q9ZDZ3 | Rickettsia prowazekii (strain Madrid E) | ACRIFLAVIN RESISTANCE PROTEIN D (AcrD) |
| Q9ZH24 | Pseudomonas aeruginosa. | Efflux pump membrane transporter |
| Q9ZHC9 | Salmonella typhimurium. | Putative cation efflux system protein SilA |
| Q9ZJQ5 | Helicobacter pylori (strain J99 / ATCC 700824) | CATION EFFLUX SYSTEM PROTEIN |
| Q9ZKN2 | Helicobacter pylori (strain J99 / ATCC 700824) | Putative cation efflux system protein |
| Q9ZLM5 | Helicobacter pylori (strain J99 / ATCC 700824) | Putative efflux transporter |
| Q9ZNG8 | Pseudomonas aeruginosa. | Efflux pump membrane transporter |
